# Nonribosomal Peptides from Marine Microbes and Their Antimicrobial and Anticancer Potential

**DOI:** 10.3389/fphar.2017.00828

**Published:** 2017-11-21

**Authors:** Shivankar Agrawal, Debabrata Acharya, Alok Adholeya, Colin J. Barrow, Sunil K. Deshmukh

**Affiliations:** ^1^Biotechnology and Management of Bioresources Division, TERI-Deakin Nano Biotechnology Centre, Energy and Resources Institute, New Delhi, India; ^2^Centre for Chemistry and Biotechnology, School of Life and Environmental Sciences, Deakin University, Waurn Ponds, VIC, Australia

**Keywords:** microbe derived-compounds, marine natural products, nonribosomal peptides, antimicrobial, anticancer

## Abstract

Marine environments are largely unexplored and can be a source of new molecules for the treatment of many diseases such as malaria, cancer, tuberculosis, HIV etc. The Marine environment is one of the untapped bioresource of getting pharmacologically active nonribosomal peptides (NRPs). Bioprospecting of marine microbes have achieved many remarkable milestones in pharmaceutics. Till date, more than 50% of drugs which are in clinical use belong to the nonribosomal peptide or mixed polyketide-nonribosomal peptide families of natural products isolated from marine bacteria, cyanobacteria and fungi. In recent years large numbers of nonribosomal have been discovered from marine microbes using multi-disciplinary approaches. The present review covers the NRPs discovered from marine microbes and their pharmacological potential along with role of genomics, proteomics and bioinformatics in discovery and development of nonribosomal peptides drugs.

## Marine ecosystem resources for new drug discovery

**Figure 1:**
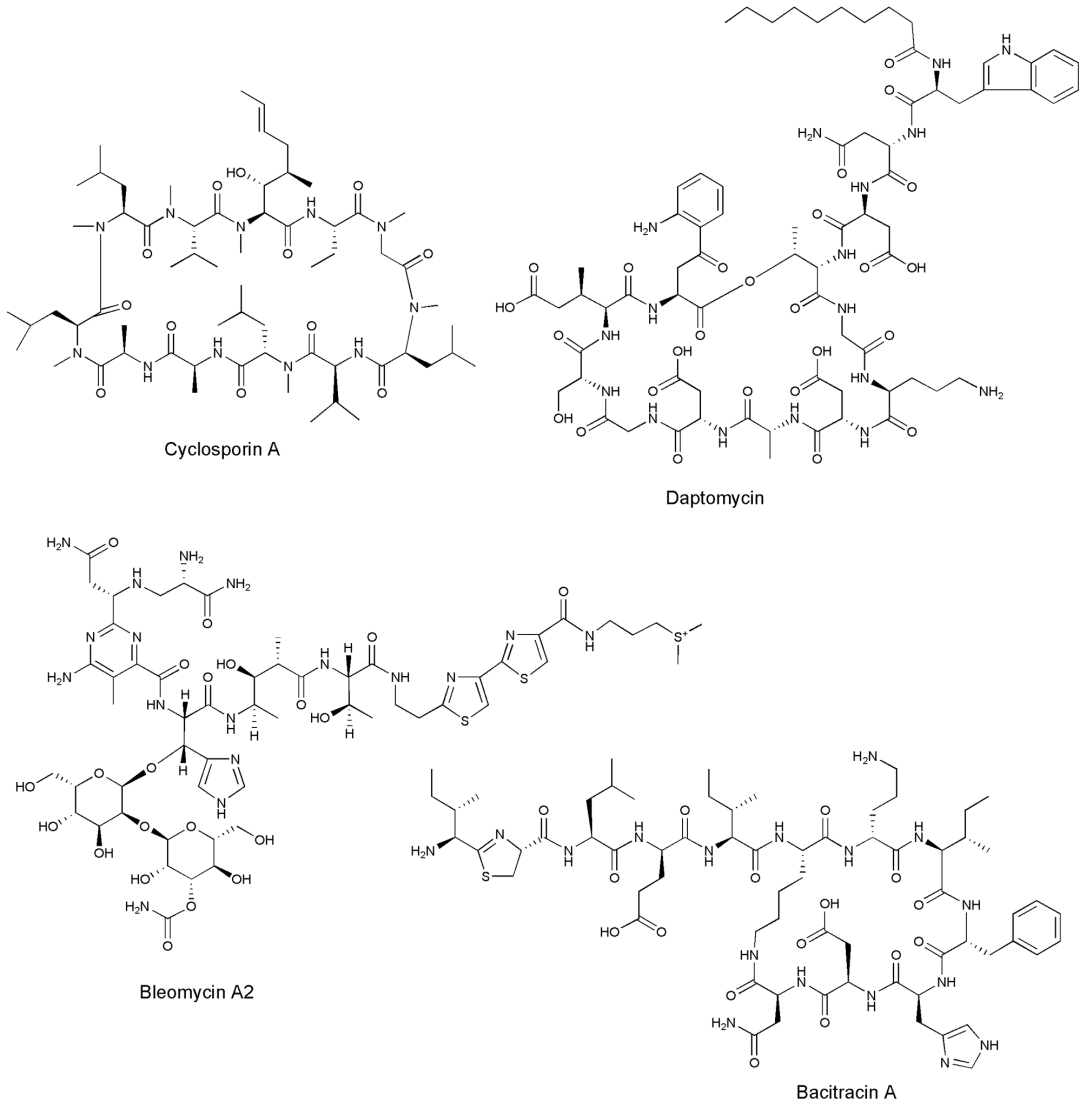
Structures of marketed NRPs.

The marine ecosystem is most complex and largest aquatic systems on earth. It includes oceans, intertidal ecology, salt marsh, lagoons, estuaries, coral reefs, mangroves, deep sea, sea floor etc. Marine ecosystem has a enormous variety of organisms that are different in their physiology and adaptations and most of the marine life is found in coastal habitats (Hedgepeth, 1957). According to the Global Biodiversity Assessment by the United Nations Environment Program, oceans consist of 178,000 marine species in 34 phyla. It is estimated that 10^2^ fungi, 10^3^ bacteria and 10^7^ viruses are likely to exist in one milliliter of seawater (Kubanek et al., 2003). Marine organisms comprise around 50% of the total biodiversity on earth. These organisms have shown remarkable contribution in the discovery and production of novel biomolecules (Jimeno et al., 2004; Vignesh et al., 2011). During 1981–2002 50% of US- FDA approved drugs are reported from either marine bioactive compounds or their synthetics analogs (Vinothkumar and Parameswaran, 2013). Cytosine arabinoside, Ara-C (anticancer) and adenine arabinoside, Ara-A (antiviral) were first discovered in the early 1950s and approved by Food and Drug Administration (US-FDA). These drugs were isolated from Caribbean sponge (*Cryptotheca crypta*), as spongouridine and spongothymidine. Blunt et al. (2015) reported more than 20,000 natural bioactive compounds have been obtained from marine environment in last 50 years (Blunt et al., 2015). Out of these 9 were approved as drugs and many of them are still in clinical trials. It is well documented that more than 50% of drugs that are in clinical use today belong to the nonribosomal peptides or mixed polyketide-NRP families (Hranueli et al., 2010; Agrawal et al., 2016; Table 1). Marine microbes contributes 70% of discovery of NRPs with antimicrobial, antiviral, cytostatic, immunosuppressant, antimalarial, antiparasitic, animal growth promoters and natural insecticides activities etc. (Vinothkumar and Parameswaran, 2013). Which makes marine microbial an important bioresource for getting NRPs with numerous pharmaceutical applications. The examples of some NPR based drugs which are now in the market are Daptomycin (antibiotics), Bleomycin (antitumor), Bacitracin (antibiotics for skin infections), Cyclosporin (antifungal and immunosuppressant drugs) (Figure 1) (Strieker et al., 2010). Norine is the first database entirely dedicated to NRPs and contains more than 1186 entries (Caboche et al., 2008, 2009). In this review we focus on antimicrobial and anticancer NRPs reported from marine microbes with their biological targets.

**Table 1:** List of some marine derived NRPs and their present status (Newman and Cragg, 2004; Fenical, 2006; Jimenez et al., 2009; Petit and Biard, 2013).

**Metabolite**	**Source**	**Pharmacological activity**	**R&D stage**
Ecteinascidin 743 (Yondelis^TM^)	*Ecteinascidia turbinate* (Sea squirt)	Anticancer	Market
Cephalosporine	*Cephalosporium acremonium* (Fungi)	Antibiotic	Market
Bengamide derivative (LAF389)	*Jaspis* sp. (Sponge)	Anticancer	Phase I
Hemiasterlin derivative (HTI-286)	*Cymbastella* sp. (Sponge)	Anticancer	Phase I
Dehydrodidemnine B (Aplidine^TM^)	*Aplidium albicans* (Tunicate)	Anticancer	Phase II
Dolastatin 10	*Dolabella auricularia* (Mollusc and Cyanobacteria)	Anticancer	Phase II
Kahalalide F	*Elysia rufescens* (Sea slug)	Antitumor	Phase II
Bryostatin 1	*Bugula neritina* (Bryozoan)	Anticancer	Phase III
Diazonamide	*Diazona angulata* (Tunicate)	Anticancer	Preclinical
Thiocoraline	*Mi Cromonospora marina* (Bacteria)	Anticancer	Preclinical
Vitilevuamide	*Didemnum cucliferum* and *Polysyncraton lithrostrotum* (Tunicates)	Anticancer	Preclinical

## Nonribosomal peptide and their bio combinatorial synthesis

An extensive literature on biosynthesis of non-ribosomal peptides is available in previous reviews (Sieber and Marahiel, 2003; Finking and Marahiel, 2004; Caboche et al., 2009; Strieker et al., 2010; Pfennig and Stubbs, 2012). Here we just summarized how NPRs are synthesized biologically, biomolecular structural architecture and enzymatic machinery of non-ribosomal peptide synthetases (NRPSs). NRPs are peptide secondary bioactive metabolites synthesized by a multi-modular enzyme complex called nonribosomal peptide synthetases (NRPSs) found only in bacteria, cyanobacteria and fungi (Matsunaga and Fusetani, 2003; Nikolouli and Mossialos, 2012). NRPs are formed from a series of enzymatic transformations employing a much more diverse set of precursors and biosynthetic reactions. NRPSs utilize both proteinogenic and nonproteinogenic amino acids (not encoded by DNA) as building blocks for the growing peptide chain (Finking and Marahiel, 2004; Felnagle et al., 2008). Moreover, these secondary bioactive metabolite peptides contain unique structural features, such as D-amino acids, N-terminally attached fatty acid chains, N- and C-methylated residues, N- formylated residues, heterocyclic elements, and glycosylated amino acids, as well as phosphorylated residues etc.; (Sieber and Marahiel, 2003). As a result, NRPs exhibit a broad spectrum of biological activities, ranging from antimicrobial to anticancer (Hur et al., 2012). The macrocyclic structure is a common feature of nonribosomally synthesized bioactive peptides, which is responsible for reduction in structural flexibility and may, therefore, constrain them into the biologically active conformation (Sieber and Marahiel, 2003; Grünewald and Marahiel, 2006).

**Figure 2:**
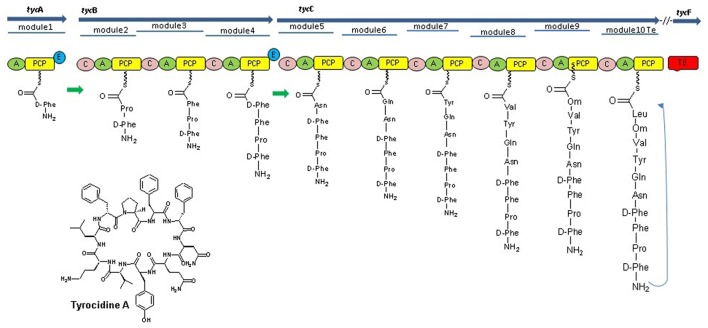
Tyrocidine biosynthesis in bacteria *B. brevis* nonribosomal peptide synthetases of tyrocidine synthesis mainly consist, three NRPSs TycA, TycB, and TycC, which contain 10 modules (TycA comprises one module, TycB three, and TycC six modules) each of those responsible for the incorporation of a cognate amino acid into the growing chain with the help of their domains. The Te domain at the last module of TycC catalyzes peptide cyclization and thereby release of the final product (Mootz et al., 2000).

**Figure 3:**
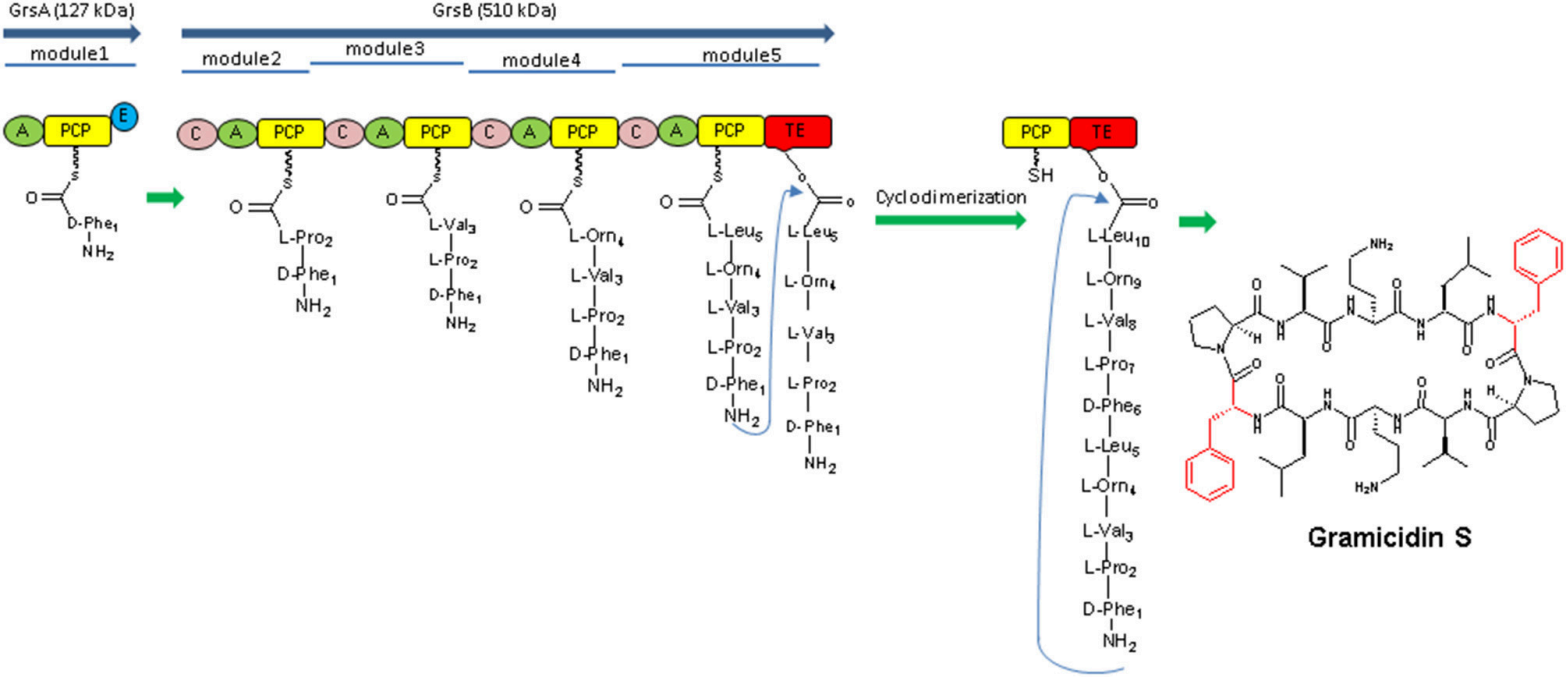
The Gramicidin S biosynthetic machinery the enzymatic assembly consists of two NRPSs (GrsA and GrsB) and their modules, respectively. Each module is responsible for the incorporation of one monomeric amino acid. The thioesterase domain (TE domain) catalyzes the dimerization of two assembled pentapeptides and subsequent cyclization, resulting in gramicidin S (Hoyer et al., 2007).

The discovery of NRPs began when Tatum and colleagues (Mach et al., 1963) provided first evidence that tyrocidine, a cyclic decapeptide produced by *Bacillus brevis*, was biosynthesized by a mechanism independent of the ribosome (Mankelow and Neilan, 2000). They found that protein synthesis in *B. brevis* was inhibited by using ribosome targeting antibiotics like chloramphenicol and chlortetracycline, however, the biosynthesis of tyrocidine was not obstructed by the same. Additional biochemical analyses demonstrated that gramicidin S, a cyclic decapeptide produced by *B.brevis*, did not include tRNA molecules or aminoacyl-tRNA-synthetases (Nikolouli and Mossialos, 2012; Figure 2). Further work by Lipmann established that the production of cyclic decapeptide, gramicidin is an ATP-dependent reaction, catalyzed by these enzymes incorporating amino acids in a two-step process by their modules and their respective domains. The first step involves release of pyrophosphate (PPi) and the second step releases adenosine monophosphate (AMP), with the end result being an amino acid covalently linked to the enzyme (Wu et al., 2003). These finding suggested that tyrocidine and gramicidin S peptide synthesis did not involve ribosomal machinery for their synthesis, which leads to discovery of the NRPs and NRPSs. These data also gave the first indication of an amino acid as a “carrier” being involved in NRPS enzymology (Felnagle et al., 2008; Condurso and Bruner, 2012; Figure 3).

**Figure 4:**
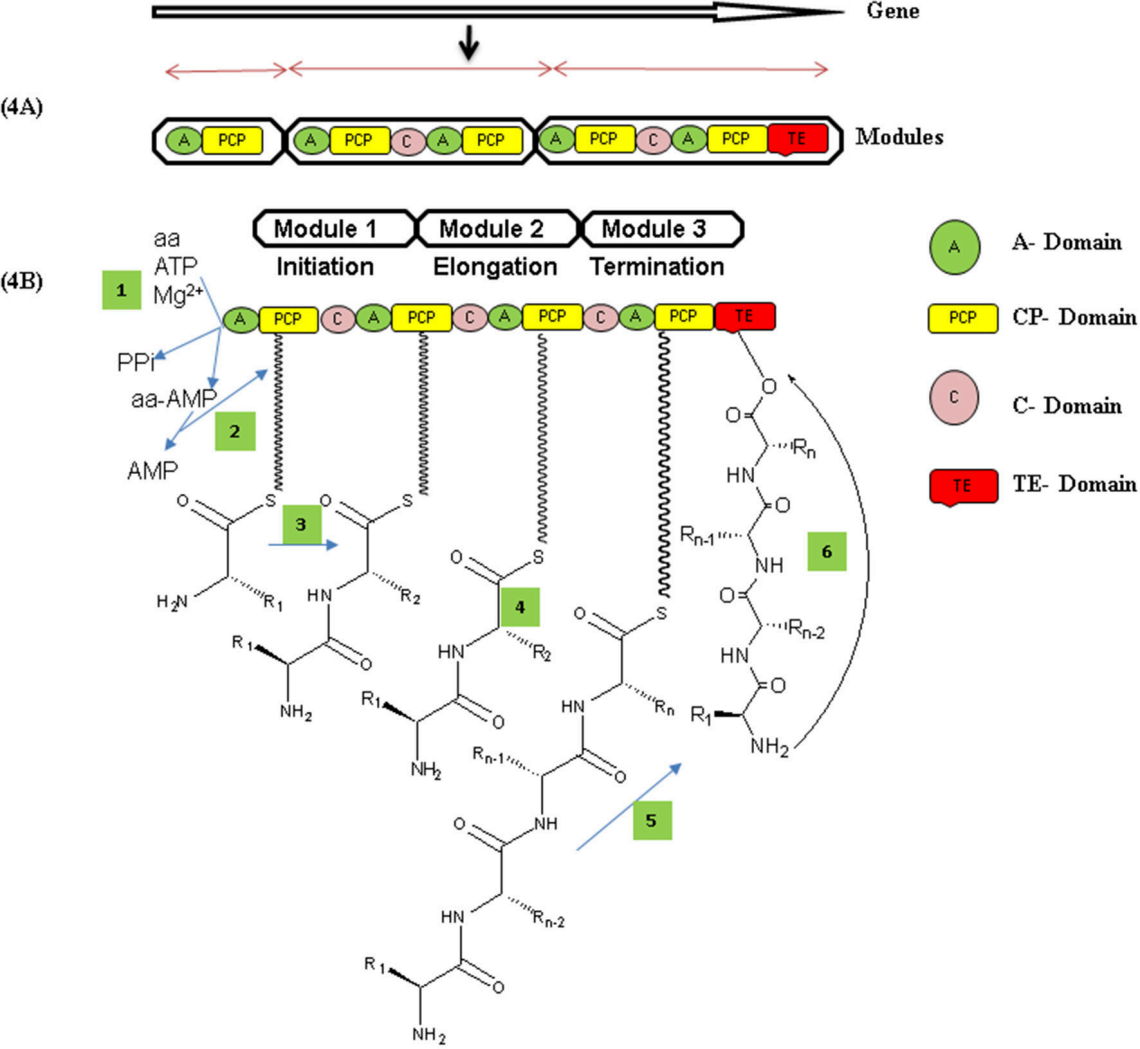
**(A)** Organization of modules and their domains in nonribosomal peptide synthetase enzyme. Each module contains their catalytic domains that catalyze activities, substrate activation (A-domain), covalent loading (CP-domain), and peptide bond formation (C-domain). The first modules always lacks a C domain and is used to initiate nonribosomal peptide synthesis, while those harboring a C-domain qualify for elongation and modules with thioesterase domains (TE) usually in the last domain, for termination of peptide product from enzyme through cyclization or hydrolysis (Prieto et al., 2012). **(B)** Mechanism of nonribosomal peptide (NRP) synthesis Adenylation domain (A) activates amino acid as aminoacyl-AMP and transfer to PCP domain which condenses coming amino acids by forming peptide bonds. Structural modifications mostly operate by epimerization domains which converts L-amino acid to D-amino acid and vice a versa. Peptide chain thus transfers to TE domain by transesterification reaction by PCP. Finally, TE domain catalyzed product release (NRPs) by either hydrolysis or macrocyclization (Condurso and Bruner, 2012).

**Figure 5:**
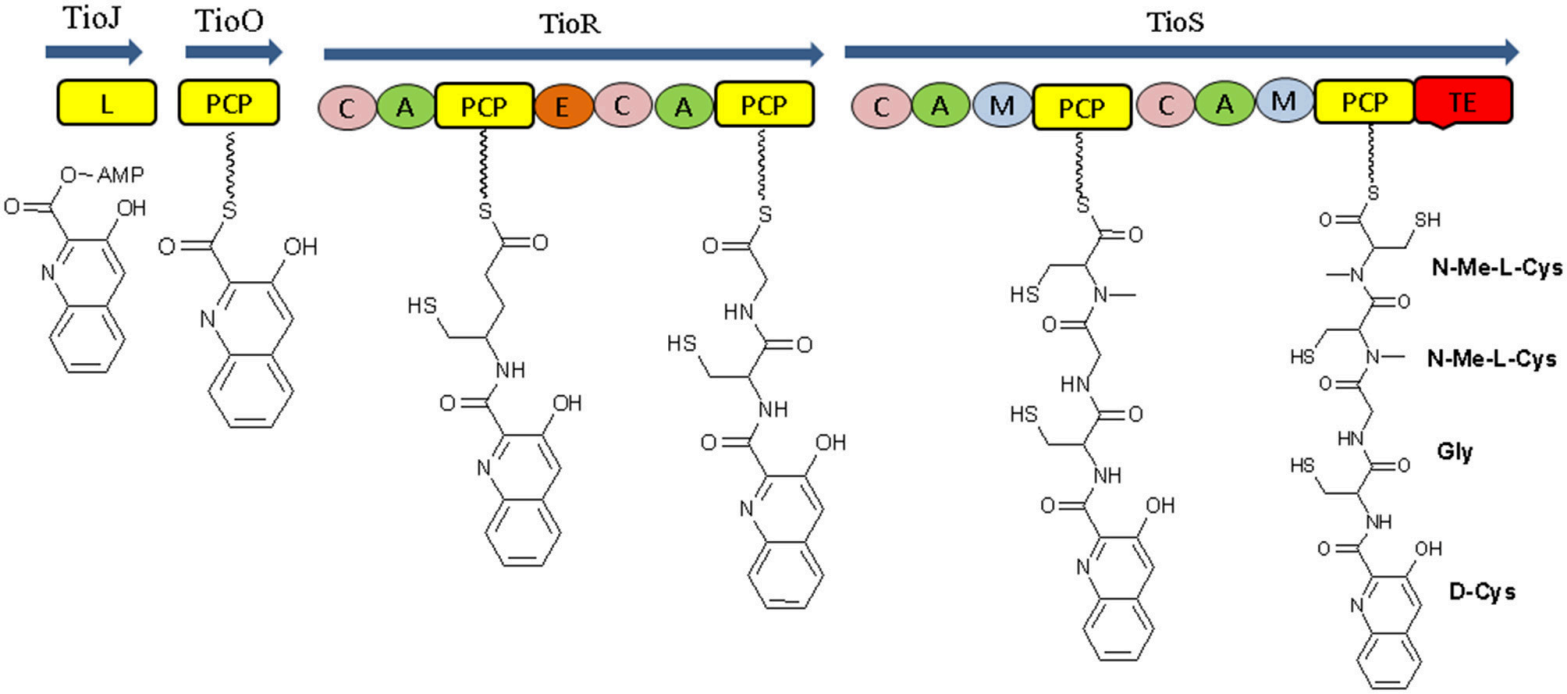
Structural organization of the thiocoraline NRPSs. L, AMP-ligase; P, peptidyl-carrier protein domain; C, condensation domain; A, adenylation domain; E, epimerization domain; M, N-methyltransferase domain; TE, thioesterase domain.

The biosynthetic study of NRP compounds is challenging if we consider their complexity and biological activities. Each nonribosomal peptide synthetase is composed of an array of distinct modular sections, each of which is responsible for the incorporation of one defined monomer into the final peptide product. Biosynthesis of a nonribosomal peptide by NRPSs involves a series of repeating reactions that are catalyzed by the coordinated actions of modules and their core catalytic domains. Each enzyme module contains three catalytic domains: adenylation domain (A), peptidyl-carrier (PCP) domain and condensation domain (C). A final peptide product released from the enzyme through cyclization or hydrolysis that takes place by thioesterase domain (TE) which is located in the final NRPSs module (Figures 4A,B; Mankelow and Neilan, 2000; Finking and Marahiel, 2004). For recent example, Thiocoraline, an anticancer nonribosomal peptide (NRP) synthesis by marine bacteria *Cromonospora marina* contains peptidic backbone of two S-methylated Lcysteine residues. S-Methylation occurs very rarely in nature, and is observed extremely rarely in nonribosomal peptide scaffold. The four modules TioJ, TioO, TioR, and TioS of thiocoraline NRPSs are responsible for the thiocoraline-backbone biosynthesis. TioR and TioS would most probably constitute the NRPSs involved in the biosynthesis of the thiocoraline, according to the colinearity of the respective modules (Figure 5; Lombó et al., 2006; Al-Mestarihi et al., 2014). The potentials of marine microbes to produce NRP's with antimicrobial and anticancer activity are reported in this review. The data referring to these activities are depicted in Tables 2–4 and the structures are given in Supplementary Materials (Figures S1–S17).

**Table 2:** Antimicrobial NRPs from marine microbes.

**Sr. No**.	**NRPs**	**Chemical architecture**	**Source**	**Biological target**	**Biological active value (MIC/IC_50_/GI_50_/ID_50_/ED_50_)**	**References**
**BACTERIA**						
1.	Bogorol A **(1)**	Linear peptide	*Bacillus laterosporus*	MRSA, VRE	2 μg/mL, 10 μg/mL	Barsby et al., 2001
2.	Nocathiacins I–III **(2–4)**	Cyclic peptide	*Nocardia* sp.	multiple-drug resistant pathogens	0.001–0.015, 0.0005-0.25, 0.002-0.06 μg/mL	Leet et al., 2003; Li et al., 2003
3.	YM- 266183 and YM- 266184 **(5–6)**	Cyclic peptide	*Bacillus cereus*	Staphylococci, Enterococci	0.05–0.2 μg/mL, 0.013–0.025 μg/mL. 0.02–0.05 μg/mL, 0.006–0.01 μg/mL	Nagai et al., 2003; Suzumura et al., 2003
4.	cyclo-(glycyl-l-seryl-l-prolyl-l- glutamyl) **(7)** and cyclo-(glycyl-l-prolyl-l-glutamyl) **(8)**	Cyclic peptide	*Ruegeria* sp.	*Bacillus subtilis*	25 and 50 μg/mL	Mitova et al., 2004
5.	Tauramamide **(9)**	Lipopeptide	*B. laterosporus*	*Enterococcus* sp.	0.1 μg/mL	Desjardine et al., 2007
6	Tetrapeptide cyclo-isoleucyl-prolyl-leucyl alanyl **(10)** and cyclo-phenylalanyl-prolyl-leucyl prolyl **(11)**	Cyclic tetra peptide	*Pseudomonas* sp.	Marine bacterial	–	Rungprom et al., 2008
7.	Unnarmicin A and C **(12–13)**	Depsipeptide	*Photobacterium* sp.	*Pseudovibrio*	7–8 μg/mL	Oku et al., 2008b
8.	Thiopeptide TP-1161 **(14)**	Cyclic peptide	*Nocardiopsis* sp.	Gram-positive bacteria	0.25–4 μg/mL	Engelhardt et al., 2010
9.	Solonamide A–B **(15–16)**	Cyclo depsipeptide	*Photobacterium halotolerans*	*S. aureus*	–	Mansson et al., 2011
10.	Fijimycin A–C **(17–19)**	Depsipeptide	*Streptomyces* sp.	MRSA	4–16 μg/mL	Sun et al., 2011
11.	Peptidolipins B–F **(20–24)**	Lipopeptide	*Nocardia* sp.	MRSA, MSSA	64 μg/mL	Wyche et al., 2012
12.	Kocurin **(25)**	Cyclic peptide	*Kocuria palustris*	MRSA	0.25 μg/mL	Martín et al., 2013
13.	Champacyclin **(26)**	Octapeptide	*Streptomyces champavatii*	*Erwinia amylovora*	25 μM	Pesic et al., 2013
14.	Ngercheumicin F–I **(27–30)**	Cyclo depsipeptide	*P. halotolerans*	*S. aureus*	5 μg/mL	Kjaerulff et al., 2013
**CYANOBACTERIA**						
15.	Lobocyclamide B **(31)**	Cyclododecapeptide	*Lyngbya confervoides*	Fluconazole-resistant *C. albicans*	–	MacMillan and Molinski, 2002
16.	Brunsvicamide A–C **(32–34)**	Cyclic hexapeptide	*Tychonema* sp.	*Mycobacterium tuberculosis* (MptpB)	7.3 μM	Müller et al., 2006
**FUNGI**						
17.	Guangomides A–B **(35–36)**	Cyclic depsipeptide	Unidentified fungus	*S. epidermis, E. durans*	100 μg/mL 100 μg/mL	Amagata et al., 2006
18.	11-O-methylpseurotin A **(37)**	Linear peptide	*Aspergillus fumigatus*	*Saccharomyces cerevisiae*	–	Boot et al., 2007
19.	Emericellamides A–B **(38–39)**	Cyclic depsipeptides	*Emericella* sp.	MRSA	3.8 and 6.0 μM	Oh et al., 2007
20.	Scopularides A–B **(40–41)**	Cyclododecapeptide	*Scopulariopsis brevicaulis*	Gram-positive bacteria	–	Yu et al., 2008
21.	Alternaramide **(42)**	Cyclic Penta depsipeptide	*Alternaria* sp. SF,5016	*B. subtilis* and *S. aureus*	(ZOI 8 mm), (ZOI 13 mm)	Kim et al., 2009
22.	Trichoderins A, A', B2 **(43–45)**	Lipopeptide	*Trichoderma* sp.	*Mycobacterium tuberculosis*	0.02–2.0 l g/mL	Pruksakorn et al., 2010
23.	Unguisin E **(46)**	Cyclic heptapeptide	*Aspergillus* sp.	Antibacterial	–	Liu and Shen, 2011
24.	Sclerotides A–B **(47–48)**	Cyclic hexapeptide	*Aspergillus sclerotiorum*	*C. albicans, Pseudomonas aeruginosa*	7.0 and 3.5 μM. nil and, 35.3 μM	Zheng et al., 2009
25.	Sclerotiotides A–K **(49–59)**	Cyclic tripeptide	*A. sclerotiorum* PT06-1	*C. albicans*	7.5, 3.8, 30, 6.7μM	Zheng et al., 2010

**Table 3:** Anticancer NRPs from marine microbes.

**Sr. No**.	**NRPs**	**Chemical architecture**	**Source**	**Biological target**	**Biological active value (MIC/IC_50_/GI_50_/ID_50_/ED_50_)**	**References**
**BACTERIA**						
1.	Mixirins A–C **(60–62)**	Cyclopeptide	*Bacillus* sp.	HCT-116 cells (colon)	0.68, 1.6, 1.3 μg/ml	Zhang et al., 2004
2.	Mechercharmycin A **(63)** Mechercharmycin B **(64)**	Cyclic peptide	*Thermoactinomyces* sp.	A549 cells (lung), Jurkat cells	4.0 × 10^−8^ M 4.6 × 10^−8^ M	Kanoh et al., 2005
3.	Bromoalterochromide A **(65)** and A' **(66)**	Chromo peptide	*Pseudoalteromonas maricaloris*	*Strongylocentrotus intermedius* eggs	–	Speitling et al., 2007
4.	Lucentamycins A–D **(67–70)**	–	*Nocardiopsis lucentensis*	HCT-116 cells (colon)	0.20 and 11 μM	Cho et al., 2007
5.	Piperazimycins A–C **(71–73)**	Cyclic hexadepsipeptide	*Streptomyces* sp.	HCT-116 cells (colon)	76 ng/mL	Miller et al., 2007
6.	Urukthapelstatin A **(74)**	Cyclic thiopeptide	*Mechercharimyces asporophorigenens*	A549 cells (lung)	12 nM	Matsuo et al., 2007
7.	Arenamides A–C **(75–77)**	Cyclo hexadepsipeptide	*Salinispora arenicola*	Tumor necrosis factor	3.7 and 1.7 μM	Asolkar et al., 2008
8.	Bacillistatins 1-2 **(78–79)**	Cyclodepsipeptide	*Bacillus silvestris*	P388 (murine lymphocytic leukemia); BXPC-3 (pancreas); MCF-7 (breast); SF-268 (CNS); NCI- H460 (lung); KM20L2 (colon); DU- 145 (prostate)	10^−4^−10^−5^ μg/mL	Pettit et al., 2009
9.	Turnagainolides A–B **(80–81)**	Cyclic peptide	*Bacillus* sp.	PI3K pathway	–	Li et al., 2011
10.	Padanamides A–B **(82–83)**	Linear tetrapeptide	*Streptomyces* sp.	Jurkat cells	20 μg/mL	Williams et al., 2011
11.	Ohmyungsamycins A–B **(84–85)**	Cyclic peptide	*Streptomyces* sp.	Cytotoxic	359–816 nM and 12.4–16.8 μM	Um et al., 2013
12.	Proximicin A–C **(86–88)**	–	*Verrucosispora* strain MG-37	AGS (gastric), HepG2 (hepatocellular), MCF 7 (breast)	0.6, 1.5, 0.25 μg/mL 0.8, 9.5, 0.7 μg/mL 7.2, 5.0, 9.0 μg/mL	Fiedler et al., 2008
**CYANOBACTERIA**						
13.	Hoiamide A **(89)**	Cyclic depsipeptide	An assemblage of *L. majuscula* and *Phormidium gracile*	Voltage-gated sodium channel	92.8 nM	Pereira et al., 2009
14.	Yanucamides A–B **(90–91)**	Cyclic depsipeptide	*Lyngbya majuscule* and *Schizothrix* sp.	Brine shrimp toxicity	5 ppm	Sitachitta et al., 2000
15.	Lyngbyabellins A **(92)**	Cyclic depsipeptide	*L. majuscula*	KB cells LoVo cells	0.03 and 0.50 μg/mL	Luesch et al., 2000
16.	Lyngbyabellin B **(93)**	Cyclic depsipeptide	*L. majuscula*	Brine shrimp (*Artemia salina*)	3.0 ppm	Milligan et al., 2000
17.	Microcyclamide **(94)**	Cyclic hexapeptide	*Microcystis aeruginosa*	P388 murine leukemia cells	24–30 μg/mL	Ishida et al., 2000
18.	Apratoxin A **(95)**	–	*L. majuscula*	KB cells and LoVo cancer cells	0.52 nM 0.36 nM	Luesch et al., 2001b
19.	Pitipeptolides A–B **(96–97)**	Cyclic depsipeptide	*L. majuscula*	LoVo cells	2.25 and 1.95 μg/mL	Luesch et al., 2001a
20.	Ulongamides A–F **(98–103)**	Cyclic depsipeptide	*Lyngbya* sp.	KB and LoVo cells	1 μM 5 μM	Luesch et al., 2002
21.	Obyanamide **(104)**	Cyclic depsipeptide	*L. confervoides*	KB cells	0.58 μg/mL	Williams et al., 2002a
22.	Malevamide D **(105)**	Linear peptide	*Symploca hydnoides*	P-388, A-549, HT-29, and MEL-28	0.7 and 0.7 nM	Horgen et al., 2002
23.	Tasiamide **(106)**	Linear peptide	*Symploca* sp.	KB and LoVo cells	0.48 and 3.47 μg/mL	Williams et al., 2002b
24.	Tasiamide B **(107)**	Linear peptide	KB	KB cells	0.8 μM	Williams et al., 2003a
25.	Guineamides A–F **(108–113)**	Cyclic depsipeptide	*L. majuscula*	Mouse neuroblastoma cells	15 and 16 μM	Tan et al., 2003b
26.	Homodolastatin 16 **(114)**	Cyclic depsipeptide	*L. majuscula*	Oesophageal Cervical cells	4.3 μg/mL 1 μg/mL	Davies-Coleman et al., 2003
27.	Lyngbyastatin 3 **(115)**	Cyclic peptide	*L. majuscula*	KB and LoVo cells	32 and 400 nM	Williams et al., 2003a
28.	Ulongapeptin **(116)**	Cyclic depsipeptide	*Lyngbya* sp.	KB cells	0.63 μM	Williams et al., 2003d
29.	Tasipeptins A–B **(117–118)**	Depsipeptide	*Symploca* sp.	KB cells	0.93 and 0.82 μM	Williams et al., 2003a
30.	Jamaicamides A–C **(119–121)**	Lipopeptide	*L. majuscula*	H-460 (lung), Neuro-2a. Sodium channel blocking	15 μM 5 μM	Williams et al., 2003d
31.	Wewakpeptins A–D **(122–125)**	Depsipeptide	*Lyngbya semiplena*	NCI-H460 neuro-2a	0.4 μM	Williams et al., 2003b
32.	Trungapeptin A–C **(126–128)**	Cyclic depsipeptide	*L. majuscula*	Ichthyotoxic Brine shrimp toxicity	6.25 ppm 10 ppm	Bunyajetpong et al., 2006
33.	Aurilides B and C **(129–130)**	Cyclic peptide	*L. majuscula*	NCI-H460 (lung), neuro-2a	0.01 and 0.13 μM	Han et al., 2006
34.	Belamide A **(131)**	Tetra peptide	*Symploca* sp.	MCF7 (breast) HCT-116 (colon)	0.74 μM	Simmons et al., 2006
35.	Lyngbyastatins 5–7 **(132–134)**	–	*Lyngbya* sp.	Potent Elastase Inhibitors	3–10 nM	Taori et al., 2007
36.	Mitsoamide **(135)**	Linear peptide	*Geitlerinema* sp.	NCI-H460 (lung)	460 nM	Andrianasolo et al., 2007
37.	Apratoxin D **(136)**	Cyclic depsipeptide	*L. majuscule* and *Lyngbya sordida*	H-460 (lung)	2.6 nM	Gutiérrez et al., 2008
38.	Apratoxin E **(137)**	–	*Lyngbya bouillonii*	HT29 (colon), HeLa (cervical),	21–72 nM	Matthew et al., 2008
39.	Dragonamide C and D **(138–139)**	Linear lipopeptide	*Lyngbya polychroa*	U2OS (osteosarcoma), HT29 (colon), IMR-32 (neuroblastoma)	56 and 59 μM 22 and 32 μM 49 and 51 μM	Gunasekera et al., 2008
40.	Coibamide A **(140)**	Cyclic depsipeptide	*Leptolyngbya* sp.	NCI 60 cancer cell	< 23 nM	Medina et al., 2008
41.	Symplostatin 4 **(141)**	Linear peptide	*Symploca* sp.	Antimitotic to H-460 (lung) neuro-2a	40 nM 29 nM	Taori et al., 2009
42.	Itralamides A–B **(142–143)**	Depsipeptide	*L. majuscula*	HEK293 cells	6 ± 1 μM	Jiménez et al., 2009
43.	Desmethoxymajusculamide C **(144)**	Cyclic and Liner peptide	*L. majuscula*	HCT-116	20 nM	Simmons et al., 2009
44.	Hantupeptin A **(145)**	Cyclic depsipeptide	*L. majuscula*	MOLT-4 (leukemia) MCF-7 (breast)	32 μM 4.0μM	Tripathi et al., 2008
45.	Desacetyl-Microcolin B **(146)**	Linear peptide	*Lyngbya cf. polychroa*	HT-29 andIMR-32 cells	14 nM 14 nM	Meickle et al., 2009
46.	Palmyramide A **(147)**	Cyclic depsipeptide	*L. majuscula*	Neuro-2a and H-460	17.2μM 39.7μM	Taniguchi et al., 2009
47.	Bisebromoamide **(148)**	Linear peptide	*Lyngbya* sp.	Protein kinase inhibitor (HeLa S3 cells)	04 μg/mL	Teruya et al., 2009
48.	Lyngbyabellin J **(149)** Lyngbyapeptin D **(150)**	Linear peptide	*L. bouillonii*	Actin-disrupting	0.041 μM	Matthew et al., 2010
49.	Grassypeptolides A–C **(151–153)**	Cyclic depsipeptide	*L. confervoides*	HT29 cells HeLa cells	1.22μM, 4.97μM, 76.7nM 1.01μM, 2.93μM, 44.6 nM	Kwan et al., 2010
50.	Hantupeptin B and C **(154–155)**	Cyclodepsipeptide	*L. majuscula*	MOLT-4 cells MCF-7 cells	0.2 μM 0.5 μM	Tripathi et al., 2010
51.	Lyngbyacyclamides A–B **(156–157)**	Cyclic peptide	*Lyngbya* sp	B16 mouse melanoma	0.7 μM	Maru et al., 2010
52.	Grassypeptolides D–E **(158–159)**	Cyclic depsipeptide	*Leptolyngbya* sp.	HeLa and neuro-2a blastoma	335 and 192 nM 599 and 407 nM	Thornburg et al., 2011
53.	Grassypeptolides F and G **(160–161)**	Cyclic depsipeptide	*L. majuscula*	Inhibit transcription (AP-1 cell)	5.2 and 6.0 μM	Popplewell et al., 2011
54.	Veraguamides A–H and J-L **(162–172)**	–	*Symploca hydnoides*	H-460 (lung)	141 nM	Mevers et al., 2011; Salvador et al., 2011
55.	Malyngamide 3 **(173)** and Cocosamide A–B **(174–175)**	Cyclic peptide	*L. majuscula*	HT-29 cells	48 μM, 24 μM, 11μM	Gunasekera et al., 2011
56.	Cyclodepsipeptide **(176–180)**	Cyclodepsipeptide	*L. majuscula*	HT-29 and MCF7	–	Montaser et al., 2010
57.	Lagunamide C **(181)**	Cyclodepsipeptide	*L. majuscula*	HT-29 and MCF7	2.1–24.4 nM	Tripathi et al., 2011
58.	Wewakamide A **(182)** Guineamide G **(183)**	Cyclic depsipeptide	*L. semiplena* and *L. majuscula*	Neuro-2a blastoma	2.7 μM	Nan et al., 2011
59.	Porpoisamide A–B **(184–185)**	Cyclic depsipeptide	*Lyngbya* sp.	HCT-116 (colon) cells U2OS (osteosarcoma) cells	25, 21μM 28, 22 μM	Meickle et al., 2011
60.	Lyngbyabellin K and L **(186–187)**, 7-epi-lyngbyabellin L **(188)** and Lyngbyabellin M-N **(189–190)**	Lipopeptide	*Moorea bouillonii*	HCT116 (colon) cells	40.9 ± 3.3 nM	Choi et al., 2012
61.	Viequeamides A–D **(191–194)**	Cyclic depsipeptide	*Rivularia* sp.	H460 (lung) cells	60 ± 10 nM	Boudreau et al., 2012
62.	Symplocin A **(195)**	Linear peptide	*Symploca* sp.	Cathepsin E inhibitor	300 pM	Molinski et al., 2012
63.	Bouillonamide **(196)**	Cyclic depsipeptide	*M. bouillonii*	Neuron 2a cells	6.0 μM	Tan et al., 2013
64.	Malyngamide 4 **(197)**	Lipopeptide	*Moorea producens*	HTCLs	–	Shaala et al., 2013
65.	Kurahyne B **(198)**	–	*Okeania* sp.	HeLa and HL60	8.1 and 9.0 μM	Okamoto et al., 2015
**FUNGI**						
66.	N-Methylsansalvamide **(199)**	Cyclic depsipeptide	*Fusarium strain* CNL-619	Tumor cell	8.3 μM	Cueto et al., 2000
67.	Dictyonamides A–B **(200–201)**	Linear dodecapeptide	Fungus K063	Kinase 4 inhibitor	16.5 μg/mL	Komatsu et al., 2001
68.	Scytalidamides A–B **(202–203)**	Cyclic heptapeptide	*Scytalidium* sp.	HCT-116 (colon)	7 and 11.0 μM	Tan et al., 2003a
69.	Trichodermamides A–B **(204–205)**	Dipeptide	*Trichoderma virens*	HCT-116 (colon)	0.32 μg/mL	Garo et al., 2003
70.	Rostratins A–D **(206–209)**	Cyclic dipeptide	*Exserohilum rostratum*	HCT-116 (colon)	8.5, 1.9, 0.76 and 16.5 μg/mL	Tan et al., 2004
71.	IB-01212 **(210)**	Cyclic depsipeptide	*Clonostachys* sp. *ESNA,A009*	LN-caP (prostrate), SK-BR3 (breast), HT29 (colon) and HELA (cervix)	10-8 M	Cruz et al., 2006
72.	Zygosporamide **(211)**	Cyclic Penta depsipeptide	*Zygosporium masonii*	SF-26 (CNS) RXF 393 (renal)	6.5 nM ≤ 5.0 nM	Oh et al., 2006
73.	Trichoderide A **(212)**	–	*Trichoderma reesei*	A375-S2 melanoma	18.5 mg/mL	Sun et al., 2006
74.	Spicellamide A–B **(213–214)**	Cyclo hexadepsipeptide	*Spicellum roseum*	Neuron 2a cells	30 μg/mL and 6.2 μg/mL	(Kralj et al., 2007
75.	1962A and 1962B **(215–216)**	Cyclic depsipeptide	Strain No. 1962	MCF-7 (breast)	100 μg/mL	Huang et al., 2007)
76.	Microsporins A–B **(217–218)**	Cyclic tetrapeptide	*Microsporum cf. gypseum*	HCT-116, Potent inhibitors of HDAC	0.6 μg/mL and 8.5 μg/mL	Gu et al., 2007
77.	Efrapeptin J **(219)**	Penta decapeptide	*Tolypocladium* sp.	HT1080	–	Hayakawa et al., 2008
78.	Cotteslosin A–B **(220–221)**	Cyclo pentapeptide	*Aspergillus versicolor*	MM418c5 (melanoma), DU145 (prostate), T47D (breast)	66 μg/mL 94 μg/mL 90 μg/mL	Fremlin et al., 2009
79.	Versicotide A–B **(222–223)**	Cyclo pentapeptide	*A. versicolor*	Anti-tumor	–	Zhou et al., 2011
80.	Fellutamide F **(224)**	Lipopeptide	*A. versicolor*	SK-MEL-2 (skin) XF498 (CNS) HCT15 (Colon)	0.67 μM 0.14 μM 0.13 μM	Lee et al., 2011
81.	Cordyheptapeptides C–E **(225–227)**	Cyclo heptapeptide	*Acremonium persicinum*	SF-268, MCF-7, and NCI-H460	2.5–12.1 μM	Chen et al., 2012
82.	Pullularin E and F **(228–229)**	–	*Bionectria ochroleuca*	L5178Y (lymphoma)	0.1 and 6.7 μg/mL	Ebrahim et al., 2012
83.	Clavatustides A–C **(230–232)**	Cyclodepsipeptide	*Aspergillus clavatus*	Hepatocellular Carcinoma Cycle Inhibitory	–	Jiang et al., 2013; Ye et al., 2014
84.	Asperterrestide A **(233)**	Cyclic tetrapeptide	*Aspergillus terreus*	U937 (carcinoma) and MOLT4 cell	6.4 and 6.2 μM	He et al., 2013
85.	Similanamide **(234)**	Cyclohexapeptide	*Aspergillus similanensis*	MCF-7 (breast), NCI-H460 (lung) and A373 (melanoma)	125 ± 0 117.50 ± 3.55 115 ± 7.07	Prompanya et al., 2015

**Table 4:** NRPs with both antimicrobial and anticancer potential from marine microbes.

**Sr. No**.	**NRPs**	**Chemical architecture**	**Source**	**Biological target**	**Biological active value (MIC/IC_50_/GI_50_/ID_50_/ED_50_)**	**References**
1.	Lajollamycin **(235)**	Spiro-β-lactone-γ-lactam	*Streptomyces nodosus*	Drug resistant gram-positive bacteria/	2–20 μg/mL	Manam et al., 2005
				murine melanoma cell line B16-F10	EC_50_ of 9.6 μM	
2.	Ariakemicins A-B **(236-237)**	Linear peptide	*Rapidithrix* sp	*S. aureus/*	0.46 μg/mL	Oku et al., 2008a
				human lung cancer cells (A549) and baby hamster kidney cells	IC_50_ values of 25 and 15 μg/mL	
3.	Ieodoglucomide A-B **(238-239)**	Glycolipopeptide	*Bacillus licheniformis*	Antibacterial/lung cancer and stomach cancer cell lines	GI_50_ values of 25.18 and 17.78 μg/mL	Tareq et al., 2012
4.	Halolitoralin A **(240)** Halolitoralins B **(241)** C **(242)**	Cyclic hexapeptide Cyclic tetrapeptide	*Halobacillus litoralis*	*Candida albicans* and *Tricophyton rubrum*/human gastric tumor	20, 30, 30 μg/mL and 25, 35, 40 μg/mL	Yang et al., 2002
5.	Mojavensin A **(243)**	Lipopeptide	*Bacillus mojavensis*	*Valsa mali, cucumerinum, and Fusarium verticillioides/*	2 mg /mL	Ma et al., 2012
				HL-60	IC_50_ of 100	
6.	Marthiapeptide A **(244)**	Cyclic peptide	*Marinactinospora thermotolerans*	SF-268 (glioblastoma), MCF-7 (breast), NCI-H460 (lung), HepG2 (hepatocarcinoma)/	0.38–0.52 μM	Zhou et al., 2012
				gram-positive bacteria		
7.	Linear heptapeptide **(245)**	Heptapeptide	*Paenibacillus profundus*	SK-MEL-28 cell /	3.07 μM/	Kalinovskaya et al., 2013
				*S. aureus, S. epidermis B. subtilis* and *Enterococcus faecium*	ZOI = 24 mm, 20 mm,28 mm, 11 mm	

## NRPs with antimicrobial potential

Antibiotic resistance in bacteria, parasites, viruses and fungi necessitates the continuous discovery of new drugs for the effective prevention and treatment of an ever-increasing range of infections caused by them (Organization, 2014). Natural products are the principal source for primary health care. Natural products are observed as a diverse group of molecules which have evolved to interact with a wide variety of protein targets for specific purposes. Also the same protein structure with little or no variation serves different purposes in different organisms. As a result, it is believed that the search for novel antimicrobial entity from natural sources will yield better results than from combinatorial chemistry and other synthetic procedures (Ngwoke et al., 2011). Here we described NRPs from marine microbial sources with antimicrobial potential.

### Bacteria

A cationic antibiotic Bogorol A **(1)** (Figure S1), displaying potent activity against both methicillin-resistant *Staphylococcus aureus* (MRSA, MIC 2 μg/mL) and vancomycin-resistant enterococcal strains (VRE, MIC 10 μg/mL) of bacteria, has been isolated from cultures of a marine *Bacillus laterosporus* collected in Papua New Guinea (Barsby et al., 2001). A new broad spectrum thiazolyl peptide antibiotic, Nocathiacins I–III **(2**–**4)** (Figure S1), was isolated from the cultured broth of *Nocardia* sp. They share structural similarities to glycothiohexide-alpha (Li et al., 2003). All compounds exhibit potent *in vitro* activity against several multiple-drug resistant pathogens with MIC of 0.001–0.015, 0.0005–0.25, 0.002–0.06 μg/mL respectively. They demonstrate excellent *in vivo* efficacy in a systemic *S. aureus* infection mouse model at PD_50_ 0.8, 0.6, 0.6 mg/kg/day respectively (Leet et al., 2003). The marine sponge *Halichondria japonica* was the source of *Bacillus cereus* which gave two cyclic thiopeptide antibiotics, YM-266183 **(5)** and YM-266184 **(6)** (Figure S1). They exhibited potent antibacterial activity against Staphylococci (MIC 0.05–0.2 μg/mL, 0.013–0.025 μg/mL) and Enterococci including multiple drug resistant strains (MIC 0.02–0.05 μg/mL, 0.006–0.01 μg/mL), whereas, they were inactive against gram-negative bacteria (Nagai et al., 2003). These structures contain thiazole and pyridine moieties and several unusual amino acids (Suzumura et al., 2003). A bacterial Ruegeria species isolated from a sponge *Suberites domuncula* (Gulf of Naples, Italy), gave two new cyclic peptides, cyclo-(glycyl-l-seryl-l-prolyl-l- glutamyl) **(7)** and cyclo-(glycyl-l-prolyl-l-glutamyl) **(8)** (Figure S1) with moderate antimicrobial activity against *Bacillus subtilis* at MIC of 25 and 50 μg/mL, respectively (Mitova et al., 2004). *B. laterosporus* PNG276 obtained from Papua New Guinea was the source of a new lipopeptide antibiotic, Tauramamide **(9)** (Figure S1). Anti-pathogenic activity against *Enterococcus* sp. were reported for tauramamide and its ethyl ester at MIC 0.1 μg/mL (Desjardine et al., 2007). *Pseudomonas* sp. separated out from the seaweed *Diginea* sp. (Ishigaki Is., Okinawa, Japan) were the source of cyclic tetrapeptides cyclo-[phenylalanyl-prolyl-leucyl-prolyl] **(10)** and cyclo-[isoleucyl-prolyl-leucyl-alanyl] **(11)** (Figure S1). The crude extract of this bacterial culture was found to inhibit the growth of other marine bacterial strains (Rungprom et al., 2008).

Unnarmicin A **(12)** and C **(13)** (Figure S2) are two depsipeptides isolated from a culture of a marine bacterium, *Photobacterium* sp. strain MBIC06485 having selective inhibitory effect on *Pseudovibrio* bacterial strains (Oku et al., 2008b). A strong antibacterial thiopeptide antibiotic TP-1161 **(14)** (Figure S2) with a rare aminoacetone moiety, have been isolated from *Nocardiopsis* sp. MICs value of TP-1161, ranging from 0.25 to 4 μg/ml for most gram-positive strain. The gene cluster for the biosynthesis of **(14)** was identified by PCR screening using degenerate primers (Engelhardt et al., 2010). Marine *Photobacterium halotolerant* yielded two novel cyclodepsipeptides Solonamide A **(15)** and B **(16)** (Figure S2) with inhibitory effect on virulence gene expression in *S. aureus* (Mansson et al., 2011). The fermentation broth of *Streptomyces* strain isolated from a marine sediment sample collected off Nasese, Fiji were the source of three new depsipeptides, Fijimycins A–C **(17–19)** (Figure S2). Fijimycins A–C were shown significant activity against three MRSA strains with MIC_100_ values between 4 and 16 μg/mL (Sun et al., 2011). Peptidolipins B–F **(20**–**24)** (Figure S2), antibacterial lipopeptides were obtained from an ascidian-derived *Nocardia* sp. Peptide **(20)** and **(23)** were moderately antibacterial against MRSA and methicillin-sensitive *S. aureus* (MSSA) (Wyche et al., 2012). A marine-derived bacterium *Kocuria palustris* was the source of a new thiazolyl peptide, Kocurin **(25)** (Figure S2). Kocurin strongly inhibits MRSA MB5393 with a MIC value of 0.25 μg/mL (Martín et al., 2013). An octapeptide, Champacyclin **(26)** (Figure S2) was isolated from three strains of *Streptomyces champavatii* (sediment, Gotland Deep and Kiel Bight, Baltic Sea and Urania Basin, Eastern Mediterranean) as an inhibitor of blight disease causing bacterium *Erwinia amylovora* (Pesic et al., 2013). Cyclodepsipeptides Ngercheumicin F–I **(27**–**30)** (Figure S2) isolated from *P. halotolerans*, inhibited quorum sensing in *S. aureus* (Kjaerulff et al., 2013).

### Cyanobacteria

Lobocyclamide B **(31)** (Figure S3) a cyclododecapeptide containing five beta-hydroxy-alpha-amino acid residues, was discovered from *Lyngbya confervoides* which was active against fluconazole-resistant *C. albicans*. The absolute stereochemistry was determined by chiral chromatography of Marfey's reaction (MacMillan and Molinski, 2002). Brunsvicamides A–C **(32**–**34)** (Figure S3), three new cyclic hexapeptides have been isolated from cyanobacterium *Tychonema* sp. Brunsvicamide C contains an N-methylated N'-formylkynurenine moiety. Brunsvicamide B selectively inhibits the *Mycobacterium tuberculosis* protein tyrosine phosphatase B (MptpB, IC_50_ 7.3 μM) (Müller et al., 2006).

### Fungi

An extraction of a saltwater culture of an unidentifiable sponge-derived fungus leads to discovering two novel cyclic depsipeptides, Guangomides A **(35)** and B **(36)** (Figure S4). Both compounds had weak antibacterial activity against *Staphylococcus epidermis* (MIC = 100 μg/mL, each) and *Enterococcus durans* (MIC = 100 μg/mL, each) (Amagata et al., 2006). A marine-derived *Aspergillus fumigatus* yielded to 11-O-methylpseurotin A **(37)** (Figure S4) (PKS/NRPS), which selectively inhibited a Hof1 deletion strain of the yeast *Saccharomyces cerevisiae* (Boot et al., 2007). Marine-derived fungus *Emericella* sp., and marine actinomycete *Salinispora arenicola* were co-cultured to induce production of Emericellamides A **(38)** and B **(39)** (Figure S4) by fungi. Emericellamides A and B displayed modest antibacterial activities against MRSA with MIC values of 3.8 and 6.0 μM, respectively (Oh et al., 2007).

The fungus *Scopulariopsis brevicaulis*, isolated from marine sponge *Tethya aurantium* was the source of two novel cyclodepsipeptides, Scopularides A **(40)** and B **(41)** (Figure S4), which were weak inhibitors of gram-positive bacteria (Yu et al., 2008). Alternaramide **(42)** (Figure S4), a cyclic Penta depsipeptide, was produced by culture of *Alternaria* sp. which was isolated from sediment, Masan Bay, S. Korea. It's showed weak antimicrobial activity against *B. subtilis* (ZOI 8 mm) and *S. aureus* (ZOI 13 mm) (Kim et al., 2009). Trichoderins A **(43)**, A1 **(44)**, and B **(45)** (Figure S4) are three new amino lipopeptides reported from marine sponge-derived fungus of *Trichoderma* sp. All trichoderins have shown potent anti-mycobacterial activity against *Mycobacterium smegmatis, Mycobacterium bovis* BCG, and *M. tuberculosis* H37Rv under aerobic and dormancy-inducing hypoxic growth conditions with MIC values in the range of 0.02–2.0 l g/mL (Pruksakorn et al., 2010).

*Aspergillus* sp. AF119 was the source of γ-aminobutyric acid containing cyclic heptapeptide Unguisin E **(46)** (Figure S4) (Liu and Shen, 2011). Marine-derived halotolerant *Aspergillus sclerotiorum* PT06-1 gave to two novel cyclic hexapeptides Sclerotides A **(47)** and B **(48)** (Figure S4) in a nutrient-limited hypersaline medium. Both of these peptides were photo inter convertible, containing anthranilic acid, dehydroamino acid units and showed moderate antifungal activity against *C. albicans*. Compound **(47)** also inhibited *P. aeruginosa* growth (Zheng et al., 2009). A halotolerant *A. sclerotiorum* PT06-1 isolated from salt sediments from the Putian Sea Salt Field, Fujian, China was the source of 11 new aspochracin-type cyclic tripeptides, Sclerotiotides A–K **(49–59)** (Figure S4). Only sclerotiotides A, B, F and I showed selective antifungal activity against *C. albicans* with MIC values of 7.5, 3.8, 30, and 6.7 μM, respectively (Zheng et al., 2010).

## NRPs with anticancer potential

Cancer is the second leading cause of death worldwide. Present therapies cause serious side effects. Therefore there is need to employ alternative concepts including natural products therapy, complementary or alternative medicine, surgery, radiation therapy used alone or in combination to the prevention of cancer (Reddy et al., 2003). Here we focus on the marine natural products specially NRPs that have been evaluated for cancer prevention.

### Bacteria

Three new cytotoxic cyclopeptides, Mixirins A–C **(60**–**62)** (Figure S5) belonging to iturin class have been isolated from marine bacterium *Bacillus* sp. obtained from the mud near the Arctic pole. All compounds inhibited the growth of human colon tumor cells (HCT-116) with IC_50_ of 0.68, 1.6, 1.3 μg/ml. (Zhang et al., 2004). A *Thermoactinomyces* specie YM3-251 have been isolated from mud (Mecherchar, Republic of Palau), which was the source of a cyclic peptide Mechercharmycin A **(63)** as well as the linear congener Mechercharmycin B **(64)** (Figure S5). Mechercharmycin A exhibited relatively strong antitumor activity against A549 cells (human lung cancer) and Jurkat cells (human leukemia) with IC_50_ value of 4.0 × 10^−8^ M and 4.6 × 10^−8^ M respectively, whereas mechercharmycin B exhibited no activity (Kanoh et al., 2005). Bromoalterochromides A and A' **(65–66)** (Figure S5), an unprecedented chromo peptide was produced by a marine *Pseudoalteromonas maricaloris* strain KMM 636 which was isolated from sponge *Fascaplysinopsis reticulata*. Chemically both of these compounds are brominated yellow colored Thr-Val-Asn-Asn-X pentapeptide lactones, where the amino group of Thr is acylated with 9-(3-bromo-4-hydroxyphenyl)-nona-2, 4,6,8-tetraenoic acid, and X is aIle and Leu, respectively. They showed moderate cytotoxic effects on developing eggs of the sea urchin *Strongylocentrotus intermedius* (Speitling et al., 2007). New 3-methyl-4-ethylideneproline-containing cytotoxic peptides, Lucentamycins A–D **(67–70)** (Figures S5, S6) have been isolated from the broth of a marine-derived actinomycete *Nocardiopsis lucentensis* (strain CNR-712). Only lucentamycins A and B showed significant *in vitro* cytotoxicity against HCT-116 human colon carcinoma with IC_50_ values of 0.20 and 11 μM, respectively (Cho et al., 2007).

Three cyclic hexadepsipeptides Piperazimycins A–C **(71**–**73)** (Figure S6) have been isolated from the fermentation broth of a *Streptomyces* sp. (sediment, Guam). The structures of these cyclic hexadepsipeptides have shown presence of rare amino acids, including hydroxyacetic acid, α-methylserine, γ-hydroxypiperazic acid, γ-chloropiperazic acid 2-amino-8-methyl-4, 6-nonadienoic acid, and 2-amino-8-methyl-4,6-decadienoic acid and were all significantly cytotoxic against multiple tumor cell lines with an average GI_50_ 76 ng/mL for each (Miller et al., 2007). The cultured mycelia of marine bacterium *Mechercharimyces asporophorigenens* (marine lake sediment, Urukthapel Island, Palau) was the source of Urukthapelstatin A **(74)** (Figure S6), a cyclic thiopeptide that displayed potent activity against a human cancer cell line panel. Urukthapelstatin A has also shown growth inhibition of human lung cancer A549 cells in dose-dependent manner with an IC value of 12 nM (Matsuo et al., 2007). The culture of *Salinispora arenicola* isolated from sea sediment (Great Astrolabe Reef, Fijiy) yielded three new cyclohexadepsipeptides, Arenamides A–C **(75**–**77)** (Figure S6). The absolute structures and configuration of these compounds were established by the spectroscopic technique. Arenamides A **(75)** and B **(76)** blocked tumor necrosis factor (TNF)-induced activation with IC_50_ values of 3.7 and 1.7 μM respectively. In addition, they also inhibited nitric oxide and prostaglandin E2 production and were moderately cytotoxic to HCT-116 cells (Asolkar et al., 2008). *Bacillus silvestris* that was isolated from a Pacific Ocean (southern Chile) crab yields two new cyclodepsipeptides, Bacillistatins 1-2 **(78**–**79)** (Figure S6) with strong anti-cancer (GI_50_ of 10^−4^-10^−5^ μg/mL) activity (Pettit et al., 2009). The epimeric cyclic peptides Turnagainolides A **(80)** and B **(81)** (Figure S6), isolated from marine *Bacillus* sp. (sediment, Turnagain Is., British Columbia, Canada), had indirect inhibitory effect on phosphatidylinositol-3-kinase (PI3K) pathway (Li et al., 2011). A *Streptomyces* sp. obtained from marine sediment produced two highly modified linear tetrapeptides, Padanamides A **(82)**, and B **(83)** (Figure S6). They inhibit cysteine and methionine biosynthesis and are cytotoxic to Jurkat cells (IC_50_ of 20 μg/mL) respectively (Williams et al., 2011). Chemical genomics was performed to discover the mode of action of compounds, which suggested that padanamide A inhibits cysteine and methionine biosynthesis.

*Streptomyces* sp. isolated from volcanic island produced new cyclic peptides Ohmyungsamycin A **(84)** and B **(85)** (Figure S7). The presence of unusual amino acid units, including N-methyl-4-methoxytrytophan, β-hydroxyphenylalanine, and N, N-dimethylvaline in compound **(84–85)** have been determined by interpretation of the NMR, UV, and IR spectroscopic and MS data. Both exhibited inhibitory activities against diverse cancer cells with IC_50_ values ranging from 359 to 816 nM and 12.4 to 16.8 μM respectively. However, compound **(84)** was more active in this regard interestingly; these compounds exhibit relatively selective anti-proliferative activity against cancer cells compared to normal cells. This may be due to the consequence of genetic background or of the biologically various characteristics between cancer and normal cells. However, the exact molecular mechanism behind the selectivity should be further investigated (Um et al., 2013). Proximicins A–C **(86**–**88)** (Figure S7) are novel aminofuran antibiotics with anticancer activity, isolated from marine strains of *verrucosispora* sp. Compounds **(86–88)** showed inhibitory activity against gastric adenocarcinoma (AGS, IG_50_ = 0.6, 1.5, 0.25 μg/mL respectively), hepatocellular carcinoma (HepG2, IG_50_ = 0.8, 9.5, 0.7 μg/mL respectively) and breast carcinoma cells (MCF 7, IG_50_ = 7.2, 5.0, 9.0 μg/mL respectively). A cell-cycle analysis in AGS cells revealed that Proximicin C produced cell arrest in the G0/G1 phase after incubation for 24 h. After 40 h, there was an increase in the number of cells in the sub-G1 phase, that is, apoptotic cells (+2.9%). It was also found that proximicin C induce upregulation of p53 and of the cyclin kinase inhibitor p21 in AGS cells (Fiedler et al., 2008).

### Cyanobacteria

An assemblage of *Lyngbya majuscula* and *Phormidium gracile* collected in Papua New Guinea produced a cyclic depsipeptide Hoiamide A **(89)** (Figure S8). The highly unusual structure of hoiamide A synthesized by mixed peptide–polyketide biosynthetic pathway showed moderate cytotoxicity to cancer cells and partial agonist of site 2 on the voltage-gated sodium channel as it produced a rapid and concentration-dependent elevation of neuronal [Na^+^] in neocortical neurons (IC_50_ = 92.8 nM) (Pereira et al., 2009). An assemblage of the marine cyanobacteria *L. majuscula* and *Schizothrix* species collected from Fiji was the source of cyclic depsipeptides Yanucamides A **(90)** and B **(91)** (Figure S8), which contain a 2, 2-dimethyl-3-hydroxyoct-7-ynoic acid moiety. Both compounds exhibited strong brine shrimp toxicity (LD_50_, 5 ppm) (Sitachitta et al., 2000). The cyclic depsipeptides named Lyngbyabellins A **(92)** (Figure S8), contain a 7,7-dichloro-2,2-dimethyl-3-hydroxyoctanoic acid moiety have been isolated from the cytotoxic fraction of *L. majuscula* collected from Guam and the Dry Tortugas National Park, Florida. Compound **(92)** have moderate cytotoxicity against human nasopharyngeal carcinoma cell line (KB cells) and human colon adenocarcinoma cell line (LoVo cells), with IC_50_ values of 0.03 and 0.50 μg/mL, respectively and also showed cellular microfilament network in A-10 cells at 0.01–5.0 μg/mL concentrations (Luesch et al., 2000). Another collection from Tortugas National Park, Florida was the source of cytotoxic and antifungal cyclic depsipeptide Lyngbyabellin B **(93)** (Figure S8). Lyngbyabellin B was toxic to brine shrimp (LD_50_ = 3.0 ppm) (Milligan et al., 2000). A marine cyanobacterium *Microcystis aeruginosa* contained the cyclic hexapeptide Microcyclamide **(94)** (Figure S8), which showed moderate cytotoxicity against P388 murine leukemia cells at 24–30 μg/mL (Ishida et al., 2000).

The cyanobacterium *L. majuscule* collected from Guam was the source of Apratoxin A **(95)** (Figure S8). This cyclodepsipeptide of mixed peptide-polyketide biogenesis exhibited *in vitro* cytotoxicity against human tumor cell lines at IC_50_ of 0.36–0.52 nM. Apratoxin A induces G1 phase cell arrest and apoptosis, which is at least particularly initiated through antagonism of FGF signaling via STAT3 (Luesch et al., 2001b). Another collection of *L. majuscule* from Guam gave two cyclic depsipeptides, Pitipeptolides A **(96)** and B **(97)** (Figure S8) with anti-mycobacterial and weak cytotoxicity against LoVo cells with IC_50_ values of 2.25 and 1.95 μg/mL, respectively. Pitipeptolides A and B also stimulated elastase activity. It is suggested that this activity is due to the presence of hydrophobic portions in the molecule (Luesch et al., 2001a). Marine cyanobacterium *Lyngbya* sp. collected from Palauan was the source of six new β-amino acid-containing cyclic depsipeptides, the Ulongamides A–F **(98**–**103)** (Figure S8). All peptides were found to be weakly cytotoxic against KB and LoVo cells with IC_50_ values of ca. 1 μM and ca. 5 μM respectively except compound Ulongamides F (Luesch et al., 2002). Examination of a *L. confervoides* collection from Saipan, Commonwealth of the Northern Mariana Islands, led to the isolation of a novel cytotoxic cyclic depsipeptide Obyanamide **(104)** (Figure S8). Obyanamide was cytotoxic against KB cells with an IC_50_ of 0.58 μg/mL. According to the results, the β-amino acid residue was found to play a critical role in the biological activities. Additionally, the ester bond along with the Ala (Thz) moiety was also essential for biological activities (Williams et al., 2002a). Malevamide D **(105)** (Figure S8), a highly cytotoxic peptide ester have been isolated from marine cyanobacterium *Symploca hydnoides* (Horgen et al., 2002). A culture *Symploca* sp. yielded Tasiamide **(106)** (Figure S8), an acyclic peptide. Tasiamide demonstrated cytotoxic activity against KB and LoVo cells with IC_50_ values of 0.48 and 3.47 μg/mL, respectively (Williams et al., 2002b). A new cytotoxic peptide Tasiamide B **(107)** (Figure S9) which contain the unusual amino acid-derived residue 4-amino-3-hydroxy-5-phenylpentanoic acid (Ahppa) have been isolated from cyanobacterium *Symploca* sp. This peptide displayed an IC_50_ value of 0.8 μM against KB cells (Williams et al., 2003b).

A Papua New Guinea collection of the marine cyanobacterium *L. majuscule* was the source of six cyclic depsipeptides, Guineamides A–F **(108**–**113)** (Figure S9). The presence of beta-amino or beta-hydroxy carboxylic acid residues in all peptides was determined using a combination of chemical manipulations as well as Marfey's method. Guineamides B and C showed moderate cytotoxicty to a mouse neuroblastoma cell line with IC_50_ values of 15 and 16 μM, respectively (Tan et al., 2003b). A new bioactive cyclic depsipeptide, Homodolastatin 16 **(114)** (Figure S9) have been isolated from *L. majuscula*, collected from Wasini Island off the southern Kenyan coast. Homodolastatin 16 showed moderate activity against oesophageal (IC_50_ = 4.3 μg/mL) and cervical cancer cell lines (IC_50_ = 1 μg/mL) (Davies-Coleman et al., 2003). An examination of an organic extract of a cyanobacterium *L. majuscula*, collected from Guam, led to the isolation of the cyclic peptide Lyngbyastatin 3 **(115)** (Figure S9). The presence of two unusual amino acid units, 3-amino-2-methylhexanoic acid (Amha) and 4-amino-2, 2-dimethyl-3-oxopentanoic acid units (Ibu) was determined by standard methods. Lyngbyastatin 3 displayed *in vitro* activity against KB and LoVo cell lines with IC_50_ values of 32 and 400 nM respectively (Williams et al., 2003a).

A collection of *Lyngbya* sp. from Palauan was the source of cytotoxic cyclic depsipeptide Ulongapeptin **(116)** (Figure S9) with an IC_50_ value of 0.63 μM against KB cells (Williams et al., 2003d). Two new depsipeptides Tasipeptins A **(117)** and B **(118)** (Figure S9) have been isolated from Palau collection of *Symploca* sp. The gross structure of all peptides **(117–118)** were determined by standard methods and was found to contain unusual amino acid-derived residue 4-amino-3-hydroxy-5-phenylpentanoic acid (Ahppa) and 3-amino-6-hydroxy-2-piperidone (Ahp) moiety respectively. Both were cytotoxic toward KB cells with IC_50_ values of 0.93 and 0.82 μM, respectively (Williams et al., 2003c). *Lyngbya majuscula* collected from Hector's Bay, Jamaica was found to contain three lipopeptides, Jamaicamides A–C **(119**–**121)** (Figure S9). Further biological investigation of the jamaicamides has revealed that they are generated by iterative hybrid PKS-NRPS assembly and exhibited cytotoxicity to both the H-460 human lung and Neuro-2a mouse neuroblastoma cell lines (IC_50_ = 15 μM for all), sodium channel blocking activity at 5 μM and ichthyotoxic activities (Edwards et al., 2004). Four new depsipeptides, Wewakpeptins A–D **(122**–**125)** (Figures S9, S10), were found cytotoxic to brine shrimp and to the NCI-H460 and neuro-2a cell lines (LC_50_ of approximately 0.4 μM). These were isolated from *Lyngbya semiplena* collected from Wewak Bay, Papua New Guinea (Han et al., 2005).

Trungapeptins A-C **(126**–**128)** (Figure S10), cyclodepsipeptides have been isolated from marine cyanobacterium *L. majuscula*. Trungapeptin A exhibited mild icthyotoxicity (6.25 ppm) and weak toxicity to brine shrimp (10 ppm) (Bunyajetpong et al., 2006). Cytotoxic cyclic peptides, Aurilides B **(129)** and C **(130)** (Figure S10) were produced by marine cyanobacterium *L. majuscula* collected from Papua New Guinea. Both aurilides B and C described to induce a dysfunction in mitochondria in NCI-H460 human lung tumor and the neuro-2a mouse neuroblastoma cell lines, with LC_50_ values between 0.01 and 0.13 μM (Han et al., 2006). A highly methylated tetrapeptide Belamide A **(131)** (Figure S10) was isolated from *Symploca* sp. (Salmedina Reef, Panama) which was shown antimitotic and cytotoxic to MCF7 breast cancer and HCT-116 cell lines (IC_50_ 0.74 μM) by microtubule disruption with structural analogy to the important linear peptides dolastatins 10 and 15 (Simmons et al., 2006). Three new analogs of dolastatin 13, Lyngbyastatins 5-7 **(132–134)** (Figure S10), have been isolated from two different collections of marine cyanobacteria, *Lyngbya* sp., from South Florida with previously reported cyclodepsipeptide somamide B. Compounds (**132**–**134**) were found to selectively inhibit elastase over several other serine proteases, with IC_50_ values for porcine pancreatic elastase ranging from 3 to 10 nM (Taori et al., 2007). A new linear peptide Mitsoamide **(135)** (Figure S10) was produced by marine cyanobacterium *Geitlerinema* sp. collected from Mitso-Ankaraha Island. Mitsoamide has shown strong activity against NCI-H460 human lung tumor cells with LC_50_ of 460 nM (Andrianasolo et al., 2007).

A Papua New Guinea collection of the marine cyanobacteria *L. majuscula* and *Lyngbya sordida* was the source of potent cytotoxic cyclodepsipeptide Apratoxin D **(136)** (Figure S10). Compound **(136)** possesses 3, 7-dihydroxy-2, 5, 8, 10, 10-pentamethylundecanoic acid as the polyketide moiety and potent *in vitro* cytotoxicity against H-460 human lung cancer cells with an IC_50_ value of 2.6 nM (Gutiérrez et al., 2008). Another collection of marine cyanobacterium *Lyngbya bouillonii* from a Guamanian was found to contain Apratoxin E **(137)** (Figure S10), which was strongly cytotoxic to several cancer cell lines at IC_50_ 21–72 nM (Matthew et al., 2008). Dragonamides C **(138)** and D **(139)** (Figures S10, S11) are linear lipopeptides isolated from the marine cyanobacterium brown *Lyngbya polychroa*. Both peptides are weak cytotoxic against several cancer cell lines with GI_50_ values of 56 and 59 μM against U2OS osteosarcoma cells, 22 and 32 μM against HT29 colon adenocarcinoma cells, and 49 and 51 μM against IMR-32 neuroblastoma cells, respectively (Gunasekera et al., 2008). Coibamide A **(140)** (Figure S11), a potent anti-proliferative highly methylated cyclic depsipeptide was isolated from a culture of *Leptolyngbya* sp. which was collected from the Coiba National Park, Panama. Compound **(140)** showed an unprecedented selectivity profile in the NCI 60 cancer cell line panel (LC_50_ < 23 nM). It causes S phase inhibition in cell cycle (Medina et al., 2008). *Symploca* sp. yielded dolastatin 10/15 hybrid linear peptide Symplostatin 4 **(141)** (Figure S11) which was shown to be antimitotic activity via microtubule depolymerization to H-460 lung cancer cells (IC_50_ = 40 nM) as well as neuro-2a neuroblastoma cells (IC_50_ = 29 nM) (Taori et al., 2009).

A culture of *L. majuscule* obtained from True Blue Bay, eastern Caribbean yielded two new depsipeptides, Itralamides A **(142)** and B **(143)** (Figure S11). Only itralamide B was found to cytotoxic to HEK293 cells IC_50_ 6 ± 1 μM (Jiménez et al., 2009). An active peptide metabolite Desmethoxymajusculamide C **(144)** (Figure S11) (DMMC) have been isolated from Fijian collection of *L. majuscule*. Both cyclic and liner version of DMMC were found potent and showed selective anti-solid tumor activity at IC_50_ = 20 nM against HCT-116 through disruption of cellular microfilament networks (Simmons et al., 2009). A Singapore collection of *L. majuscule* has been shown to produce cyclodepsipeptide, Hantupeptin A **(145)** (Figure S11) with cytotoxicity to MOLT-4 leukemia cells (IC_50_ 32 μM) and MCF-7 breast cancer cells (IC_50_ 4.0 μM) (Tripathi et al., 2008). Chemical investigation of *Lyngbya cf. polychroa* resulted in isolation of a linear peptide desacetylmicrocolin B **(146)** (Figure S11), was a growth inhibitor of HT-29 (IC_50_ 14 nM) and IMR-32 cells (IC_50_ 14 nM) (Meickle et al., 2009).

Palmyramide A **(147)** (Figure S11), a cyclic depsipeptide found to block sodium channel in neuro-2a cells (IC_50_ 17.2 μM) and modest cytotoxicity to H-460 cells (IC_50_ 39.7 μM) and was isolated from from a Palmyra Atoll Collection of the marine cyanobacterium *L. majuscule* (Taniguchi et al., 2009). *Lyngbya* sp. was the source of a potent cytotoxic peptide Bisebromoamide **(148)** (Figure S11) which potentially inhibit protein kinase and is cytotoxic to HeLa S3 cells with an IC_50_ value of 0.04 μg/mL (Teruya et al., 2009). An examination of an organic extract of the cyanobacterium *L. bouillonii*, collected from Guam, led to the isolation of cytoskeletal actin-disrupting and cytotoxic (IC_50_ = 0.041 μM) Lyngbyabellin J **(149)** and a linear modified peptide, lyngbyapeptin D **(150)** (Figure S11) (Matthew et al., 2010). Marine cyanobacterium *L. confervoides* gave bis-thiazoline containing cyclic depsipeptides, Grassypeptolides A–C **(151-153)** (Figure S12). All grassypeptolides cause G1 phase cell cycle arrest in HT29 (IC_50_ = 1.22, 4.97 μM, 76.7 nM) and HeLa cell lines (IC_50_ = 1.01, 2.93 μM, 44.6 nM) (Kwan et al., 2010). Hantupeptins B **(154)** and C **(155)** (Figure S12), two cytotoxic cyclodepsipeptides were obtained from a marine cyanobacterium *L. majuscule* collected from Pulau Hantu Besar, Singapore. Compound **(154)** gave an IC_50_ of 0.2 μM against MOLT-4 and 0.5 μM against MCF-7 cancer cell lines however compound **(155)** showed moderate cytotoxicity against the MOLT-4 and MCF-7 cancer cell lines with IC_50_ values of 3.0 μM and 1.0 μM, respectively (Tripathi et al., 2010). Marine cyanobacteria *Lyngbya* sp. was the source of novel cyclic peptides Lyngbyacyclamides A **(156)** and B **(157)** (Figure S12) which moderately inhibited the growth of B16 mouse melanoma cells (IC_50_ of 0.7 μM) (Maru et al., 2010).

The marine cyanobacterium *Leptolyngbya* sp. collected from the SS Thistlegorm shipwreck in the Red Sea offered two cyclic depsipeptides, Grassypeptolides D **(158)** and E **(159)** (Figure S12). Both of these peptides were cytotoxic to HeLa (IC_50_ = 335 and 192 nM, respectively) and mouse neuro-2a blastoma cells (IC_50_ = 599 and 407 nM, respectively) (Thornburg et al., 2011). Bis-thiazoline-containing cyclic depsipeptides Grassypeptolides F **(160)** and G **(161)** (Figure S12) which contains rare β-amino acid, extensive N-methylation and a large number of d-amino acids was isolated from an extract of Palauan cyanobacterium *L. majuscule*. Both **(160)** and **(161)** were found to have moderate inhibitory activity against the transcription factor AP-1 (IC_50_ = 5.2 and 6.0 μM, respectively) (Popplewell et al., 2011). An examination of an organic extract of the cyanobacterium *Symploca cf. hydnoides* sampled from Cetti Bay, Guam, led to the isolation of the eleven new peptides Veraguamides A-G **(162–168)**, Veraguamide H **(169)** and J–L **(170–172)** (Figure S12). Veraguamide A showed potent cytotoxicity to H-460 human lung cancer cell line at LD_50_ = 141 nM whilst the others were weak inhibitors. Their structures were elucidated by combining various techniques in spectroscopy, chromatography, and synthetic chemistry (Mevers et al., 2011; Salvador et al., 2011).

Three new cyclic peptides Malyngamide 3 **(173)** and Cocosamides A **(174)** and B **(175)** (Figure S13) have been isolated from the lipophilic extract of marine cyanobacteria *L. majuscula* collected from Cocos Lagoon, Guam and were found to modestly cytotoxic to HT-29 cells with IC_50_ value of 48, 24, and 11 μM respectively (Gunasekera et al., 2011). *L. majuscula* (Piti Bomb Holes, Guam) was the source of proline rich unusual cyclic depsipeptide Pitiprolamide **(176)** (Figure S13). Further investigation yielded four more peptides Pitipeptolides C–F **(177–180)** (Figure S13). All peptides were found moderately cytotoxic against two HTCLs, however, pitipeptolides C–F were more active against *M. tuberculosis* and *B. cereus* as compared to compound **(176)** (Montaser et al., 2010). The marine cyanobacterium *L. majuscule* collected from western lagoon of Pulau Hantu Besar, Singapore was the source of cyclodepsipeptide Lagunamide C **(181)** (Figure S13). Lagunamide C exhibited potent cytotoxic activity against HTCLs with IC_50_ values ranging from 2.1 to 24.4 nM, antimalarial activity against *Plasmodium falciparum* (IC_50_ 0.29 μM) and weak anti-swarming activity against *P. aeruginosa* (Tripathi et al., 2011).

A collection of marine cyanobacterium *L. semiplena* and *L. majuscule* from Papua New Guinea led to isolation of the cyclic depsipeptides Wewakamide A **(182)** and Guineamide G **(183)** (Figure S13) were respectively. Both of these peptides displayed potent toxicity against brine shrimp and only guineamide G showed cytotoxicity to a mouse neuroblastoma cell line with LC_50_ values of 2.7 μM (Nan et al., 2011). A *Lyngbya* sp. collected in Florida Keys was found to contain epimeric cyclic depsipeptides Porpoisamide A **(184)** and B **(185)** (Figure S13) which was weakly cytotoxic to HCT-116 (IC_50_ = 25, 21 μM respectively) and osteosarcoma U2OS cells (IC_50_ = 28, 22 μM respectively) (Meickle et al., 2011). *Moorea bouillonii* (Strawn Is., Palmyra Atoll, Central Pacific Ocean) gave five lipopeptides Lyngbyabellin K **(186)** and L **(187)**, 7-epi-lyngbyabellin L **(188)** and Lyngbyabellin M **(189)** and N **(190)** (Figure S13). Of note, cyclic metabolites **(189)** and **(190)** possess rare monochlorination on the 3-acyloxy-2-methyloctanoate residue, whereas unusual N, N-dimethylvaline containing lyngbyabellin N was strongly cytotoxic to HCT116 colon cancer cell line (IC_50_ = 40.9 ± 3.3 nM) (Choi et al., 2012). Viequeamides are novel 2, 2-dimethyl-3-hydroxy-7-octynoic acid (Dhoya)-containing cyclic depsipeptides isolated from a shallow subtidal collection of a “button” cyanobacterium *Rivularia* sp. (Vieques, Puerto Rico). The absolute structures and configurations of major components Viequeamide A-D **(191–194)** (Figures S13, S14) of the mixture were established by spectroscopic technique. However, viequeamides B–F were not separated out and only viequeamide A showed high cytotoxicity against H460 human lung cancer cells at IC_50_ 60 ± 10 nM (Boudreau et al., 2012). A new N, N-dimethyl-terminated linear peptide Symplocin A **(195)** (Figure S14) was produced by Bahamian collection of cyanobacterium *Symploca* sp. The absolute configuration of symplocin A was done by chiral-phase HPLC of the corresponding 2-naphthacyl esters. Symplocin A showed potent inhibitory effect on protease enzyme cathepsin E with IC_50_ 300 pM (Molinski et al., 2012). A collection of tropical marine cyanobacterium, *M. bouillonii*, from New Britain, Papua New Guinea resulted in isolation of a novel cytotoxic cyclic depsipeptide, Bouillonamide **(196)** (Figure S14). Compound **(196)** which contains two unique polyketide-derived moieties, a 2-methyl-6-methylamino-hex-5-enoic acid residue and a unit of 3-methyl-5-hydroxy-heptanoic acid have shown mild toxicity against neuron 2a mouse neuroblastoma cells with IC_50_ 6.0 μM (Tan et al., 2013). A new lipopeptides, Malyngamide 4 **(197)** (Figure S14) as a moderate inhibitor of several HTCLs have been isolated from marine cyanobacterium *Moorea producens* collected from the Red Sea, Saudi Arabia (Shaala et al., 2013). The marine cyanobacterium *Okeania* sp. collected from the coast near Jahana, Okinawa, was the source of Kurahyne B **(198)** (Figure S14). It showed growth inhibition against HeLa and HL60 cells, with IC_50_ values of 8.1 and 9.0 μM, respectively (Okamoto et al., 2015).

### Fungi

A culture of marine fungi *Fusarium* CNL-619 was the source of a new cyclic depsipeptide N-Methylsansalvamide **(199)** (Figure S15), which showed weak *in vitro* cytotoxicity against NCI human tumor cell lines (GI_50_ 8.3 μM) (Cueto et al., 2000). An unidentified fungus isolated from the red alga, *Ceradictyon spongiosum* (Okinawa) have been shown to produce two linear dodecapeptides, Dictyonamides A **(200)** and B **(201)** (Figure S15). Only the compound **(200)** showed inhibitory effect on cyclin-dependent kinase 4 with IC_50_ value of 16.5 μg/mL (Komatsu et al., 2001). A culture of marine fungus, *Scytalidium* sp., collected from Bahamas was the source of two new cyclic heptapeptides Scytalidamides A **(202)** and B **(203)** (Figure S15) and both compounds displayed moderate cytotoxicity to the HCT-116 cell line *in vitro* with IC_50_ values of 2.7 and 11.0 μM, respectively (Tan et al., 2003a). A strain of *Trichoderma virens* was isolated from ascidian *Didemnum molle* and from the surface of a green alga of genus *Halimeda* from Papua New Guinea, which was the source of two modified dipeptides Trichodermamides A **(204)** and B **(205)** (Figure S15). Trichodermamide B has showed significant *in vitro* cytotoxicity against HCT-116 cells (colon carcinoma) with an IC_50_ of 0.32 μg/mL (Garo et al., 2003). A fungal strain *Exserohilum rostratum* associated with a marine cyanobacterial mat produced four moderately cytotoxic cyclic dipeptides Rostratins A–D **(206–209)** (Figure S15). The structures and absolute configurations of peptides were determined by two-dimensional NMR techniques and Mosher method respectively. Compounds **(206–209)** exhibit activity against colon carcinoma (HCT-116) with IC_50_ values of 8.5, 1.9, 0.76, and 16.5 μg/mL, respectively (Tan et al., 2004).

A new cytotoxic cyclodepsipeptide, IB-01212 **(210)** (Figure S15) was produced by filamentous fungus *Clonostachys* sp., ESNA-A009 isolated from an unidentified Japanese sponge. IB-01212 was potent cytotoxic to several human tumor cell lines which includes LN-caP (prostrate), SK-BR3 (breast), HT29 (colon), and HELA (cervix) cell lines with GI_50_ (growth inhibition) in order of 10^−8^ M (Cruz et al., 2006). A culture of *Zygosporium masonii* isolated from a marine cyanobacterium afforded a new cyclic Penta depsipeptide, Zygosporamide **(211)** (Figure S15), which had significant cytotoxicity in the NCI's 60 cell line panel, CNS cancer cell line SF-268 (GI_50_ = 6.5 nM) and the renal cancer cell line RXF 393 (GI_50_ ≤ 5.0 nM) (Oh et al., 2006). *Trichoderma reesei* isolated from China, Lianyungang collection of sea mud produced moderately cytotoxic Trichoderide A **(212)** (Figure S15) (Sun et al., 2006). Two new cyclohexadepsipeptides, Spicellamide A **(213)** and Spicellamide B **(214)** (Figure S15) obtained from fermentation of *Spicellum roseum* (Ectyplasia perox, Dominica), exhibited cytotoxicity to neuroblastoma cells with IC_50_ value of 30 and 6.2 μg/mL respectively (Kralj et al., 2007). Two new cyclic depsipeptides 1962A, cyclo-(d-Leu-Gly-l-Tyr-l-Val-Gly-S-O-Leu) **(215)**, and 1962B, cyclo-(d-Leu-Gly-l-Phe-l-Val-Gly-S-O-Leu) **(216)** (Figure S15) have been isolated from the fermentation broth of the mangrove endophytic fungus isolated from the leaf of *Kandelia candel*. Compound **(215)** only showed activity against human breast cancer MCF-7 cells with an IC_50_ value of 100 μg/mL (Huang et al., 2007).

Two new cyclic tetrapeptides Microsporins A **(217)** and B **(218)** (Figure S15) with potent inhibitors of histone deacetylase (HDAC), cytotoxic to HCT-116 cells (IC_50_ 0.6 and 8.5 μg/mL) was isolated from the marine-derived fungus *Microsporum gypseum* (Gu et al., 2007). A Penta decapeptide, Efrapeptin J **(219)** (Figure S16), a down-regulator of the molecular chaperone GRP78 have been isolated from *Tolypocladium* sp. (sea mud, *Aomori Prefecture*, Japan) (Hayakawa et al., 2008). An Australian marine isolate of *Aspergillus versicolor* (MST-MF495) offered two cyclo pentapeptides, Cotteslosins A **(220)** and B **(221)** (Figure S16) (Fremlin et al., 2009). Two new cyclic pentapeptides, Versicotides A **(222)** and B **(223)** (Figure S16) came from marine fungus strain ZLN-60, identified as *A. versicolor* (Zhou et al., 2011). A Cytotoxic lipopeptide Fellutamide F **(224)** (Figure S16) have been isolated from the sponge-derived fungus *A. versicolor* with cytotoxicity to several human tumor cells, especially SK-MEL-2 (skin, IC_50_ 0.67 μM), XF498 (CNS, IC_50_ 0.14 μM) and HCT15 (Colon, IC_50_ 0.13 μM) (Lee et al., 2011). Fermentation extract of the marine-derived fungus *Acremonium persicinum* SCSIO 115 resulted in the discovery of three new cyclo heptapeptides, Cordyheptapeptides C–E **(225–227)** (Figure S16) with cytotoxicity against SF-268, MCF-7, and NCI-H460 tumor cell line with IC_50_ values ranging from 2.5 to 12.1 μM (Chen et al., 2012). Chemical investigation of endophytic fungus *Bionectria ochroleuca* isolated from the inner leaf tissues of the plant *Sonneratia caseolaris* (Sonneratiaceae) from Hainan Island (China), lead to discover two new peptides, Pullularins E and F **(228-229)** (Figure S16). Both compounds exhibited moderate cytotoxic activity against the mouse lymphoma cells (L5178Y) with EC_50_ values ranging between 0.1 and 6.7 μg/mL (Ebrahim et al., 2012). An unusual anthranilic acid dimer and a d-phenyllactic acid residue containing cyclodepsipeptides Clavatustides A–C **(230–232)** (Figure S16) were discovered from cultured mycelia and broth of *Aspergillus clavatus* C2WU isolated from *Xenograpsus testudinatus* and suppressed proliferation of HTCLs (Jiang et al., 2013; Ye et al., 2014). *Aspergillus terreus* SCSGAF0162 gave a new cytotoxic (HTCLs) and antiviral (H1N1 and H3N2) cyclic tetrapeptide, Asperterrestide A **(233)** (Figure S16). Which was cytotoxic toward human carcinoma U937 and MOLT4 cell lines with IC_50_ values of 6.4 and 6.2 μM, respectively, and also showed inhibitory effects on the influenza virus strains A/WSN/33 (H1N1) and A/Hong Kong/8/68 (H3N2) with IC_50_ values of 15 and 8.1 μM, respectively (He et al., 2013). A new cyclohexapeptide, Similanamide **(234)** (Figure S16) was isolated from sponge-associated fungus *Aspergillus similanensis* KUFA 0013 with weak anticancer activity (Prompanya et al., 2015).

Marine microorganisms have been recognized as one of the most promising groups of organisms from which novel pharmacologically active molecules, with potential benefits against cancer, can be isolated. Recently, several compounds have been emerged as templates for the development of novel anticancer drugs. However the mechanisms implicated in the cytotoxicity of these compounds in tumor cell lines are still largely overlooked but several studies point to an implication in apoptosis. For instance, several compounds were found to inhibit cell growth in a large variety of cancer cell lines, the pathways by which cancer cells are inhibited are still poorly elucidated. In some cases, compounds were found to induce cell death by activation of the apoptotic process; nevertheless the mechanisms underlying the apoptosis still need more investigations. Some compounds were found to create an imbalance in cellular redox potential, with mitochondria representing a central role in the process. However, more studies are needed in order to clarify it. Cell cycle is another disturbed process, mainly due to disruption of the microtubules and actin filaments; however there are only a few studies connecting marine NRPs with alterations in cell cycle and more studies are needed in order to clarify the involvement of these compounds in the process. Even membrane sodium channels can establish interactions with the compounds, revealing its potentially important role in the observed effects. In summary, more investigations are needed in order to clarify the specific targets and the mechanisms that are behind cancer cell cytotoxicity, namely the involvement of the apoptotic process by the implication of functional genomics.

## NRPs with both antimicrobial and anticancer potential

Lajollamycin, **(235)** (Figure S17) a nitro-tetraene spiro-β-lactone-γ-lactam antibiotic have been isolated from marine actinomycete *Streptomyces nodosus*. *In vitro* lajollamycin inhibited the growth of the murine melanoma cell line B16-F10 with an EC_50_ of 9.6 μM and also displayed antimicrobial activity against both drug resistant and sensitive gram-positive bacteria with MIC 2–20 μg/mL (Manam et al., 2005). Two unusual linear hybrid polyketide-nonribosomal peptide antibiotics, Ariakemicins A-B **(236**–**237)** (Figure S17) have been isolated from the fermentation broth of the marine gliding bacterium *Rapidithrix* sp., (Ariake Inland Sea, Japan). These antibiotics contain threonine, two omega-amino-(omega-3)-methyl carboxylic acids with diene or triene units, and delta-isovanilloylbutyric acid and selectively inhibited Gram-positive bacteria among which *S. aureus* was the most affected (MIC 0.46 μg/mL) and were slightly cytotoxic to human lung cancer cells (A549) and baby hamster kidney cells with IC_50_ values of 25 and 15 μg/mL respectively (Oku et al., 2008a). Glycolipopeptides Ieodoglucomide A **(238)** and B **(239)** (Figure S17) have been isolated from marine-derived bacterium *Bacillus licheniformis* (sediment, Ieodo Reef, S. Korea). Compounds **(238)** and **(239)** displayed moderately *in vitro* antimicrobial activity. However, ieodoglucomide B also displayed cytotoxic activity against lung cancer and stomach cancer cell lines with GI_50_ values of 25.18 and 17.78 μg/mL, respectively (Tareq et al., 2012).

Halolitoralin A (a cyclic hexapeptide) **(240)** (Figure S17), Halolitoralin B and C, two cyclic tetrapeptides **(241**–**242)** (Figure S17) were isolated from the marine sediment-derived *Halobacillus litoralis* YS3106. All three cyclopeptides show surprisingly simple architectures with highly repeated residue units. Compounds **(241–242)** have shown antifungal activity against two human fungi *Candida albicans* and *Tricophyton rubrum* with MIC of 20, 30, 30 μg/mL and 25, 35, 40 μg/mL respectively. In addition, these three cyclopeptides showed moderate anti-human gastric tumor activities *in vitro* (with a cell line of BGC) (Yang et al., 2002). Bioactivity-guided fractionation from the fermentation broth of *Bacillus mojavensis* B0621A (Pearl oyster Pinctada martensii, Weizhou Is., South China Sea) was the source of antifungal iturinic lipopeptide Mojavensin A **(243)** (Figure S17). The Marfey's analysis of mojavensin A provides the novel peptide backbone of L-Asn1, D-Tyr2, D-Asn3, L-Gln4, L-Pro5, D-Asn6, L-Asn7 and an anteiso-type of the saturated β-fatty acid side chain. Compound 243 also inhibited the growth of HL-60 with IC_50_ of 100 (Ma et al., 2012). A new sequential tristhiazole-thiazoline-containing cyclic peptide, Marthiapeptide A **(244)** (Figure S17), have been isolated from a culture of the deep South China Sea-derived strain *Marinactinospora thermotolerans* SCSIO 00652. Marthiapeptide A exhibited inhibition against a panel of gram-positive bacteria, with MIC values ranging from 2.0 to 8.0 μg/mL, and displayed strong cytotoxic activity against a panel of human cancer cell lines with IC_50_ values ranging from 0.38 to 0.52 μM (Zhou et al., 2012). A new linear glyceryl acid derived heptapeptide (Glyceryl-D-leucyl-D-alanyl-D-leucyl-D-leucyl-L-valyl-D-leucyl-D-alanine, **(245)** (Figure S17), were produced by the culture of marine deep sediment strain *Paenibacillus profundus* Sl 79. The compound **(245)** was cytotoxic to SK-MEL-28 cell line (IC_50_ = 3.07 μM after 72 h) and also inhibited the growth of *S. aureus* (ZOI 24 mm), *S. epidermis* (ZOI 20 mm), *B. subtilis* (ZOI 28 mm) and *Enterococcus faecium* (ZOI 11 mm) (Kalinovskaya et al., 2013).

## Role of genomics, proteomics and bioinformatics in discovery and development of nonribosomal peptides drugs

The non-ribosomal peptides (NRPs) are an essential source of chemical diversity for drug discovery and development. At present, there are more than 1,164 different non-ribosomal peptides known in public database (NCBI) which consists of over 500 unique monomers, including both proteinogenic and non-proteinogenic L- and D-amino acids as well as carboxylic acids and amines (Caboche et al., 2010). Due to great structural diversity (linear, cyclic and branched or other complex primary structures) these complex secondary metabolites had impact on all therapeutic area, as making them suitable to be used as clinical agents. However, such potential NRPs often need to be modified to improve their clinical properties and/or bypass resistance mechanisms (Bush, 2012). For instance, FDA approved Oritavancin has been developed by using semi-synthesis strategy from Vancomycin for treatment of drug resistant skin infections (Markham, 2014). Indeed, modification in the nucleotide sequence of a natural NRPS gene or combining modules of different NRPSs may potentially lead them to be more effective with unique pharmacological activity. However, this requires in-depth understanding of both the assembly line and the resulting products. Over the last few decades several bioengineering approaches have been developed to increase the yields of NRPs and generating modified peptides with altered bioactivity or improved physicochemical properties (Winn et al., 2016). Earlier, biosynthetic generation of novel NRPs analogs focused on precursor directed biosynthesis (PDB) or mutasynthesis. In PDB, a wild-type organism is provided with modified or synthetic amino acids with the prospect that the substrate specificity of the relevant NRPS shall be flexible enough to allow addition of the modified precursors into the final peptide. However, mutasynthesis is the exact opposite. The modified substrates are fed to an engineered organism which lacks the enzyme(s) required for the biosynthesis of a specific natural precursor, so that a modified substrate or precursor analog may be effectively incorporated (Weist et al., 2004). These methods are important because they generate natural product analogs rapidly.

In earlier reviews many examples of precursor directed biosynthesis of NRPs are available (Thiericke and Rohr, 1993). Other methods being adopted for the production of new nonribosomal peptides is engineering of precursor supply *in vivo* or introducing tailoring enzymes from other pathways with new glycosylation, halogenation and sulfation enzymes being applied outside of their native clusters to create structural diversity. Although it's similar to precursor directed biosynthesis, it focuses mainly on endogenous biosynthesis rather than exogenous feeding. The introduction of halogen unit into NRP scaffolds has been a common target. For example, when the enzyme PrnA (a favin-dependent tryptophan-7-halogenase) from *Pseudomonas fuorescens* Pf-5 was expressed alongside the NRPS genes for the uridyl peptide antibiotic pacidamycin, produced by *Streptomyces coeruleorubidus*, a new halogenated analog was generated (Roy et al., 2010). Using such a technique enduracidin analogs have been produced by altering halogenase in wild-type *Streptomyces fungicidicus* (Yin et al., 2010). An alternative but complicated strategy has also been developed to generate novel NRPs. It exchanges NRPS subunit, module, and domain of the core peptide itself. Initially, this method was applied by Cubist Pharmaceuticals for the development and marketing of nonribosomal peptide antibiotic daptomycin, first natural product antibiotic that gained approval for clinical use in over 30 years (Baltz et al., 2006). Unfortunately, Cubist Pharmaceuticals failed to identify any daptomycin variants with better antibacterial property than parent daptomycin. Another route that has also been explored which involves modifying the length of the peptide chain by deletion or insertion of one or more modules (Mootz et al., 2002; Butz et al., 2008). A recent study indicates that the introduction of individual or combined point mutations in the binding pocket of an NRPS adenylation domain generates new diversity of NRPs (Han et al., 2012).

A latest technique called heterologous expression offer considerable promise especially for natural hosts which are slow growing, genetically difficult to handle, unculturable, or even unknown. The transfer of biosynthetic genes from the original microbial organisms to more amenable heterologous host bacteria is more amenable to large-scale fermentation production would overcome the limitation of procurement of the drug from the ocean (which is currently limited to expensive aquaculture or field harvesting) and ensure supply (Ongley et al., 2013). The gene cluster responsible for polyketide epothilone (a potential anticancer agent) biosynthesis in the myxobacterium *Sorangium cellulosum* was cloned and completely sequenced by Tang et al. (2000). Concomitant expression of these genes in the actinomycete *Streptomyces coelicolor* produced epothilones A and B (Tang et al., 2000). After this heterologous expression system portends a plentiful supply of this medically relevant agent. Similarly A novel gene (amyZ) encoding a cold-active and salt-tolerant α-amylase (AmyZ) was cloned from marine bacterium *Zunongwangia profunda* (MCCC 1A01486) and the protein was expressed in *Escherichia coli* (Qin et al., 2014). The Ptchi19 gene of the marine *Pseudoalteromonas tunicata* CCUG 44952T was cloned and expressed in *E.coli* (García-Fraga et al., 2015). A new κ-carrageenase gene from marine bacterium Zobellia sp. ZM-2 was cloned and expressed in *E.coli* (Liu et al., 2013). Heterologous expression of the barbamide biosynthetic gene cluster from the marine cyanobacterium *Moorea producens* in the terrestrial also led to the production of a new barbamide congener 4-O-demethylbarbamide (Kim et al., 2012). The biosynthetic pathway for bacitracin was successfully transferred from *Bacillus licheniformis* to the related species *B. subtilis* (Eppelmann et al., 2001). The polyketide biosynthesis pathway for the marine-derived telomerase inhibitor griseorhodin A was productively transferred to *Streptomyces lividans* from an environmental *Streptomyces* isolate (Li and Piel, 2002). Ugai et al. (2016) got success in heterologous expression of the cryptic gene cluster found in *A. solani* to obtain a marine-derived antifungal agent didymellamide B from the *A. oryzae* transformant introducing PKS–NRPS, *trans*-ER, and P450 genes *asolSCA* (Ugai et al., 2016). Likewise many other successful examples are available in literature (Fortman and Sherman, 2005; Luo et al., 2016; Winn et al., 2016).

All studies presented above for production of novel NRPs and engineering NRPS assembly lines in the native host are laborious having low throughput and low yield. Recent advances in genome sequencing, gene synthesis, metabolomics and bioinformatics revolutionized the process of NRPS engineering. In-silico based bioprospecting of available microbial genome sequences gives us a quick look at the hidden biosynthetic capacity of natural products in the microbial species. Several active as well as silent enzymes have been identified in fungal and bacterial genomes which are involved in the biosynthesis of NRPs. The corresponding secondary metabolites of these enzymes have not been identified to date (Brakhage, 2013; Doroghazi and Metcalf, 2013). Various powerful computational algorithms and tools have been developed to analyze BGC and to determine whether they are likely to encode unique compounds (Medema and Fischbach, 2015). Comprehensive ranges of software tools are available for identification of BGC in genome sequences. These tools are generally divided into two categories: high-confidence/low-novelty and low-confidence/high-novelty. High-confidence/low-novelty includes tools such as CLUSEAN13, ClustScan14, np.searcher15, SMURF16 and antiSMASH (Medema et al., 2011). These tools analyze Hidden Markov Models (HMMs) with manually curated cutoffs to identify signature genes or domains that are highly specific for known classes of biosynthetic pathways. Such strategies give a quick and reliable interpretation of NRPs gene cluster of a single strain from its genome sequence. Low-confidence/high-novelty mainly focuses on the identification of new BGC types by applying three approaches; namely pattern-based mining, phylogenetic mining and comparative genomic mining. These further include Cluster Finder, EvoMining, and Algorithim, respectively. Tools for identification of BGCs with respect to metagenomes include PCR-based sequence-tag and the shotgun assembly approach. The sequence-tag approach identifies clones from selected harbor pathways in metagenomic libraries by amplifying known biosynthetic domains using PCR. This is particularly useful for identifying variants of known pathway types. This has been also used to identify gene clusters encoding close relatives of molecule such as rapamycin, teicoplanin, and thiocoraline (Owen et al., 2013). However, the tag-based approach can be used to find entirely new molecules that are produced by known BGC classes, especially when coupled with phylogenomic tools such as NaPDoS (Ziemert et al., 2012). These tools find application in identifying domains that represent new areas of the extant biosynthetic diversity. A range of systems have been developed to predict the substrate specificities of NRPS adenylation domains (Röttig et al., 2011; Prieto et al., 2012). Tools such as NP.searcher and antiSMASH individual monomer predictions are then combined to give a rough idea of the core scaffold of a nonribosomal peptide. Simultaneously, advancement in mass spectrometry gives efficient dereplication for analysis of small-molecule products of biosynthetic pathways (Nielsen and Larsen, 2015). NRPQuest algorithm uses molecular networking approach to identify potential gene clusters for observed tandem mass spectra of NRPs (Mohimani et al., 2014). The search database for NRPquest generates all possible orders of NRPS assembly lines within each detected NRP BGC hence, predicting the amino acids encoded by each of its module using NRPSPredictor2 (Röttig et al., 2011). A chemoinformatic based library and informatic search strategy for natural products (iSNAP) has also been doveloped for true nontargeted dereplication across a spectrum of nonribosomal peptides and within natural product extracts (Ibrahim et al., 2012). It is clear that the tools and techniques discussed above have accelerated the discovery and development of novel NRPs with desirable biological activities.

## Conclusion and future prospects of marine derived nonribosomal peptides

Marine chemicals often possess quite novel structures which in turn lead to pronounced biological activity and novel pharmacology. The study of such chemicals, therefore, is a very promising endeavor. There are three parallel branches in marine natural products chemistry: marine biomedicinals, marine chemical ecology and marine toxins. Integration of these three fields of study gives marine natural products chemistry its exclusive character and vigor. The search among marine chemicals for medically useful agents involves two steps, discovering the type of biological activity and studying the pharmacological mechanism of the activity. It is now clear that efforts to date in marine natural product chemistry have largely focused on easily collected microorganisms and their major metabolites, and while there has been a recent shift to, as detailed above, minor metabolites present in very small quantities are a challenge for analytical and biological evaluations.

As has been demonstrated in this review, the potential for nonribosomal peptides from marine as sources and/or leads to drugs that have pharmacological effects (i.e., cancer and anti-infective) is only now being realized. Combining enzyme technology and solid phase peptide synthesis, it is possible to generate a vast variety of unique peptides composed of non-proteinogenic amino acids with unique pharmacological and biotherapeutic potential. It is possible that in coming years at least one or more marine derived novel nonribosomal peptide will enter into commerce as a drug. In concluding, the huge ranges of nonribosomal peptides that have so far been identified from marine resources frequently have no comparable equivalent in terrestrial organisms. The work by (predominately) young investigators on the many aspects of nonribosomal peptides (like biosynthesis) in the commensal and/or symbiotic microbes associated with these invertebrates, or in the microbes isolated from shallow and deep sediments will increase the numbers of nonribosomal peptides from marine for further work. The marine system has hardly been scratched as yet!

## Author contributions

SA collected the available bibliographic information and wrote the manuscript. AA and CB conceived the study. SD and DA reviewed the collected information critically.

### Conflict of interest statement

The authors declare that the research was conducted in the absence of any commercial or financial relationships that could be construed as a potential conflict of interest.
