# Supplementary material for: Nonribosomal Peptides from Marine Microbes and Their Antimicrobial and Anticancer Potential
Source: Front Pharmacol. 2017 Nov 21;8:828. doi: 10.3389/fphar.2017.00828 (PMC5702503; doi:10.3389/fphar.2017.00828)
Supplement: Supplementary file 1 [file DataSheet1.docx]

**Figure S1: Structures of antimicrobial nonribosomal peptides from marine bacteria (1-11)**

**Figure S2: Structures of antimicrobial nonribosomal peptides from marine bacteria (12-30)**

**Figure S3: Structures of antimicrobial nonribosomal peptides from marine cyanobacteria (31-34)**

**Figure S4: Structures of antimicrobial nonribosomal peptides from marine fungi (35-59)**

**Figure S5: Structures of anticancer nonribosomal peptides from marine bacteria (60-68)**

**Figure S6: Structures of anticancer nonribosomal peptides from marine bacteria (69-83)**

**Figure S7: Structures of anticancer nonribosomal peptides from marine bacteria (84-88)**

**Figure S8: Structures of anticancer nonribosomal peptides from marine cyanobacteria (89-106)**

**Figure S9: Structures of anticancer nonribosomal peptides from marine cyanobacteria (107-122)**

**Figure S10: Structures of anticancer nonribosomal peptides from marine cyanobacteria (123-138)**

**Figure S11: Structures of anticancer nonribosomal peptides from marine cyanobacteria (139-150)**

**Figure S12: Structures of anticancer nonribosomal peptides from marine cyanobacteria (151-172)**

**Figure S13: Structures of anticancer nonribosomal peptides from marine cyanobacteria (173-193)**

**Figure S14: Structures of anticancer nonribosomal peptides from marine cyanobacteria (194-198)**

**Figure S15: Structures of anticancer nonribosomal peptides from marine fungi (199-218)**

**Figure S16: Structures of anticancer nonribosomal peptides from marine fungi (119-234)**

**Figure S17: Structures of nonribosomal peptides with both antimicrobial and anticancer potential (235-245)**
